# Supplementary material for: Global economic exposure to climate change amplified by spatially compounding climate extremes
Source: Nat Commun. 2026 Mar 3;17:3385. doi: 10.1038/s41467-026-70127-6 (PMC13066631; doi:10.1038/s41467-026-70127-6)
Supplement: Supplementary file 1 — Supplementary Information [file 41467_2026_70127_MOESM1_ESM.pdf]

Supplementary Information for article

Global Economic Exposure to Climate Change Amplified by Spatially Compounding Climate Extremes

Bianca Biess[1], **Lukas Gudmundsson**[1], **Sonia I. Seneviratne**[1]

[1]Institute for Atmospheric and Climate Science, Department of Environmental Systems Science, ETH Zurich, Switzerland

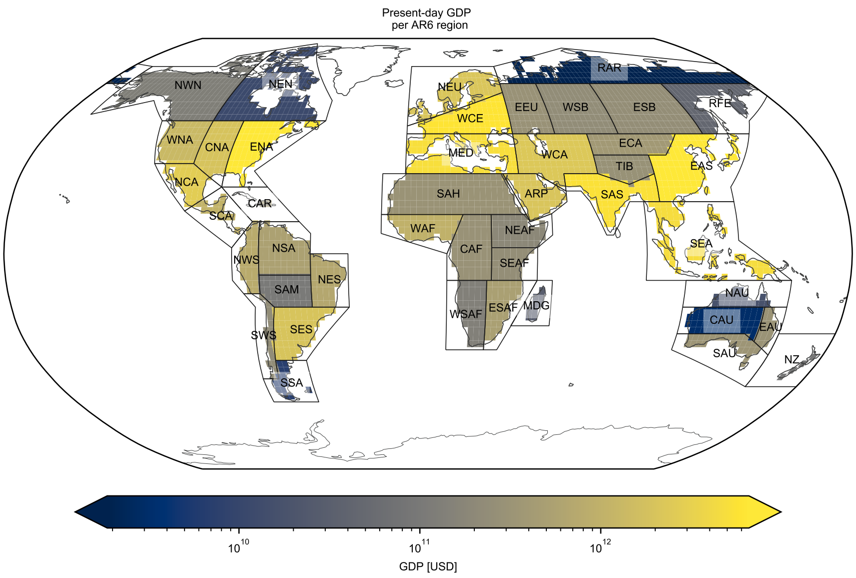

NAF: Northern Africa	WAS: West Asia	NZ: New Zealand	NEU: Northern Europe
SAH: Sahara	CAS: Central Asia	NSA: Northern South America	WCE: Western and Central Europe
WAF: Western Africa	TIB: Tibetan Plateau	NCA: North Central America	EEU: Eastern Europe
CAF: Central Africa	EAS: East Asia	SCA: South Central America	MED: Mediterranean
NEAF: Northeastern Africa	ARP: Arabian Peninsula	CAR: Caribbean	GIC: Greenland/Iceland
SEAF: Southeastern Africa	SAS: South Asia	AMZ: Amazon	NEN: Northern North America
SWAF: Southwestern Africa	SEA: Southeast Asia	NEB: Northeast Brazil	WNA: Western North America
SCAF: South Central Africa	NAU: Northern Australia	CBZ: Central Brazil	CNA: Central North America
RAR: Russian Arctic	CAU: Central Australia	SAM: South America	ENA: Eastern North America
WSB: Western Siberia	SAU: Southern Australia	SWS: Southwest South America	
ESB: Eastern Siberia	EAU: Eastern Australia	SES: Southeast South America	
RFE: Russian Far East	WAU: Western Australia	SSA: Southern South America	

Supplementary Figure 1: Regions as defined in the Sixth Assessment Report of the Intergovernmental Panel on Climate Change (AR6 regions [1]), along with their nominal gross domestic product (GDP) values for 2001–2020, based on Wang and Sun [2].

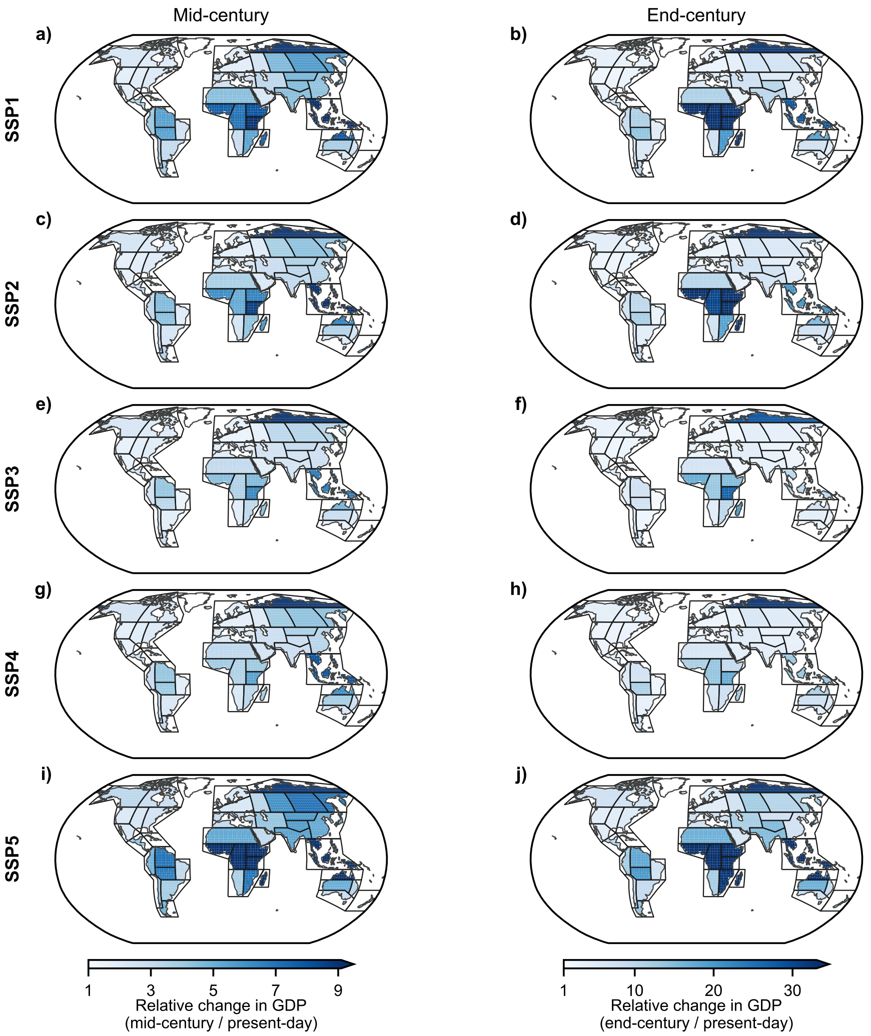

Supplementary Figure 2: Relative change in regional gross domestic product (GDP) for the mid-century (2041-2060) and end-century (2081-2100) horizons relative to present-day (2001–2020) values under five different Shared Socioeconomic Pathway (SSP) narratives. SSP1 (a: mid-century, b: end-century), SSP2 (c: mid-century, d: end-century), SSP3 (e: mid-century, f: end-century), SSP4 (g: mid-century, h: end-century), and SSP5 (i: mid-century, j: end-century). GDP is based on the dataset of Wang and Sun[2].

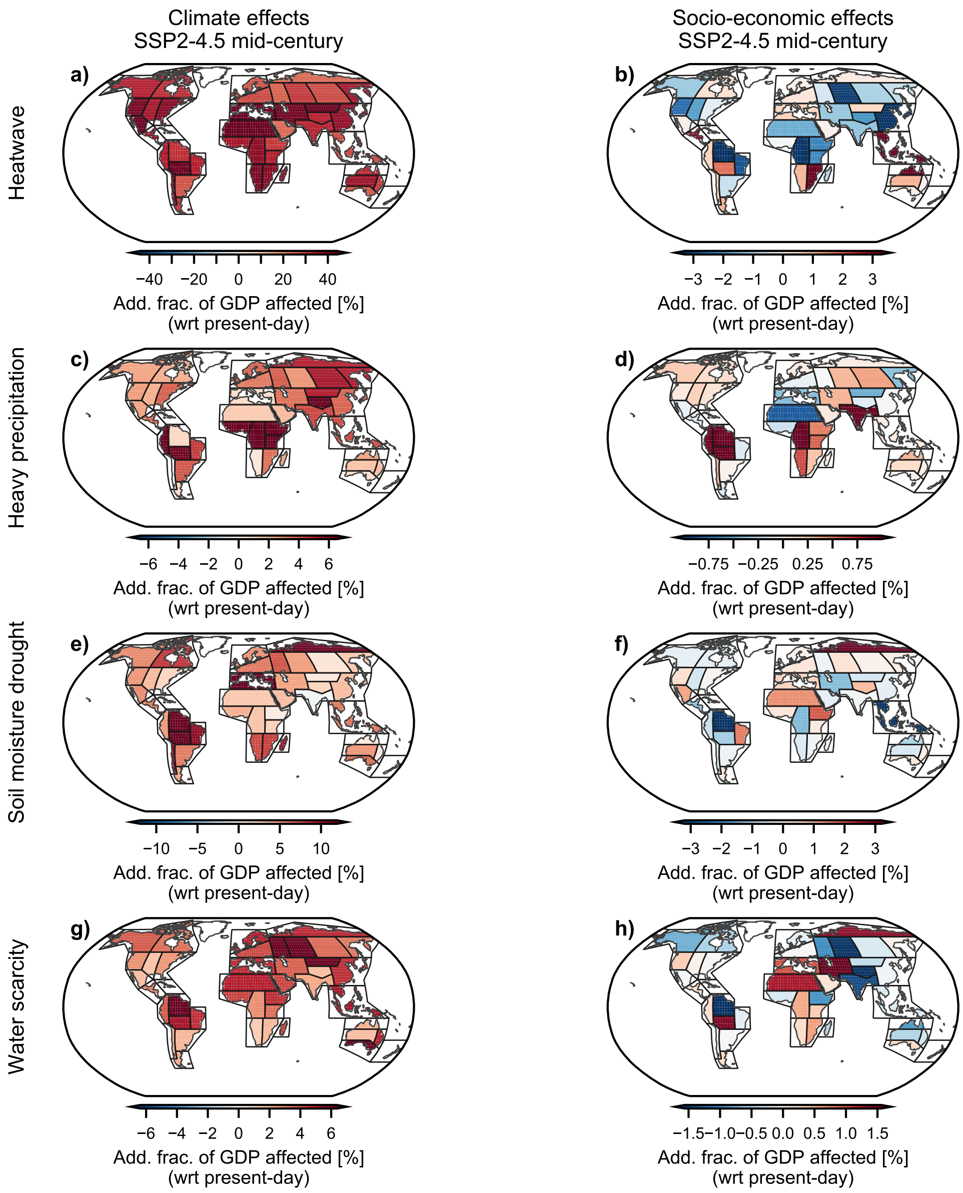

Supplementary Figure 3: Regional additional fraction of gross domestic product (GDP) affected by heatwaves (panels a–b), heavy precipitation (panels c–d), soil moisture droughts (panels e–f), and water scarcity (panels g–h) under Shared Socioeconomic Pathway 2 combined with a radiative forcing level of 4.5 W m^{-2} (SSP2-4.5) in the mid-century (2041-2060) period. Panels a, c, e, and g show GDP exposure driven by climate change, whereas panels b, d, f, and h show GDP exposure driven by socio-economic growth.

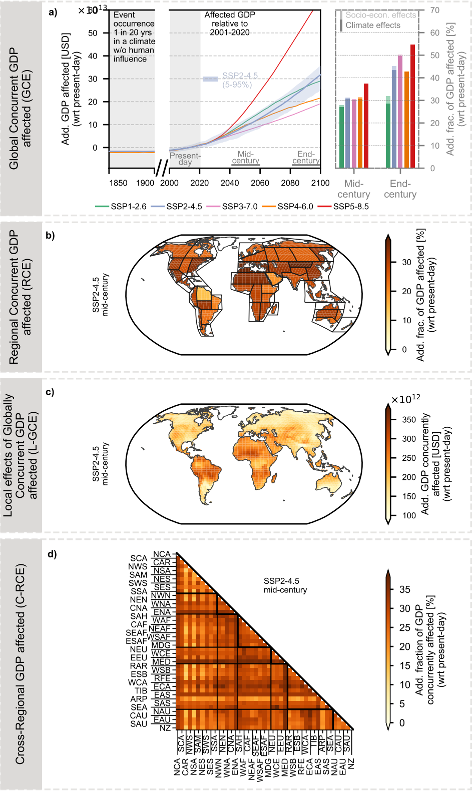

Supplementary Figure 4: Exposure of gross domestic product (GDP) to spatially compounding climate extremes, relative to present-day climate conditions (2001–2020). Grid cells may be affected by a single extreme-event type—heatwaves, heavy precipitation, water scarcity, or soil-moisture drought—or by any combination of these four hazards. **a) Global Concurrent GDP affected by Extremes (GCE):** Trajectories of additional GCE under five Shared Socioeconomic Pathway (SSP) narratives combined with their respective Radiative Concentration Pathway (RCP). The bar plot shows the additional fraction of GDP affected by at least one extreme-event type in the mid-century (2041–2060) and end-century (2081–2100) periods for each scenario, distinguishing between GDP exposure driven by climate change (present-day GDP becoming newly affected) and exposure driven by economic growth (additional GDP beyond 2020 levels becoming affected). **b) Regional Concurrent GDP affected (RCE):** Regional distribution of additional GDP affected by at least one extreme-event type under the SSP2 scenario combined with a radiative forcing level of 4.5 W m^{-2} (SSP2-4.5) in the mid-century period (2041–2060), relative to present-day climate conditions (2001–2020). **c) Additional Local Effects of Global Concurrent GDP affected (L-GCE):** Spatial distribution of additional L-GCE for at least one extreme-event type under the SSP2–4.5 scenario in the mid-century period (2041–2060), relative to present-day climate conditions (2001–2020). **d) Cross-Regional Concurrent GDP affected (C-RCE):** Additional C-RCE for at least one extreme-event type under the SSP2–4.5 scenario in the mid-century period (2041–2060), relative to present-day climate conditions (2001–2020).

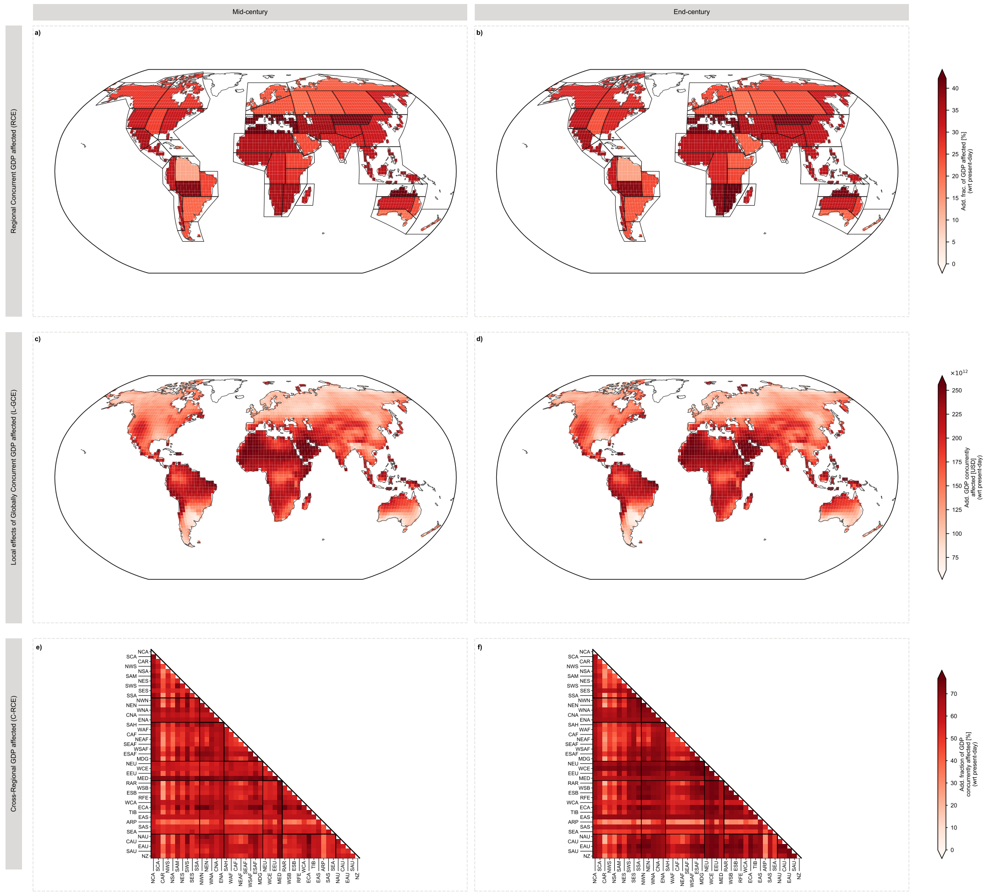

Supplementary Figure 5: Exposure of gross domestic product (GDP) to spatially compounding heatwaves under the Shared Socioeconomic Pathway 1 (SSP1) narrative combined with a radiative forcing level of 2.6 W m^{-2} (SSP1-2.6), shown relative to present-day climate conditions (2001–2020). **Regional Concurrent GDP affected (RCE):** Regional distribution of additional GDP affected by heatwaves for (a) mid-century (2041-2060) and (b) end-century (2081-2100) horizons. **Local Effects of Global Concurrent GDP affected (L-GCE):** Regional distribution of additional GDP affected by heatwaves for (c) mid-century and (d) end-century horizons. **Cross-Regional Concurrent GDP affected (C-RCE):** Additional C-RCE affected by heatwaves for (e) mid-century and (f) end-century horizons.

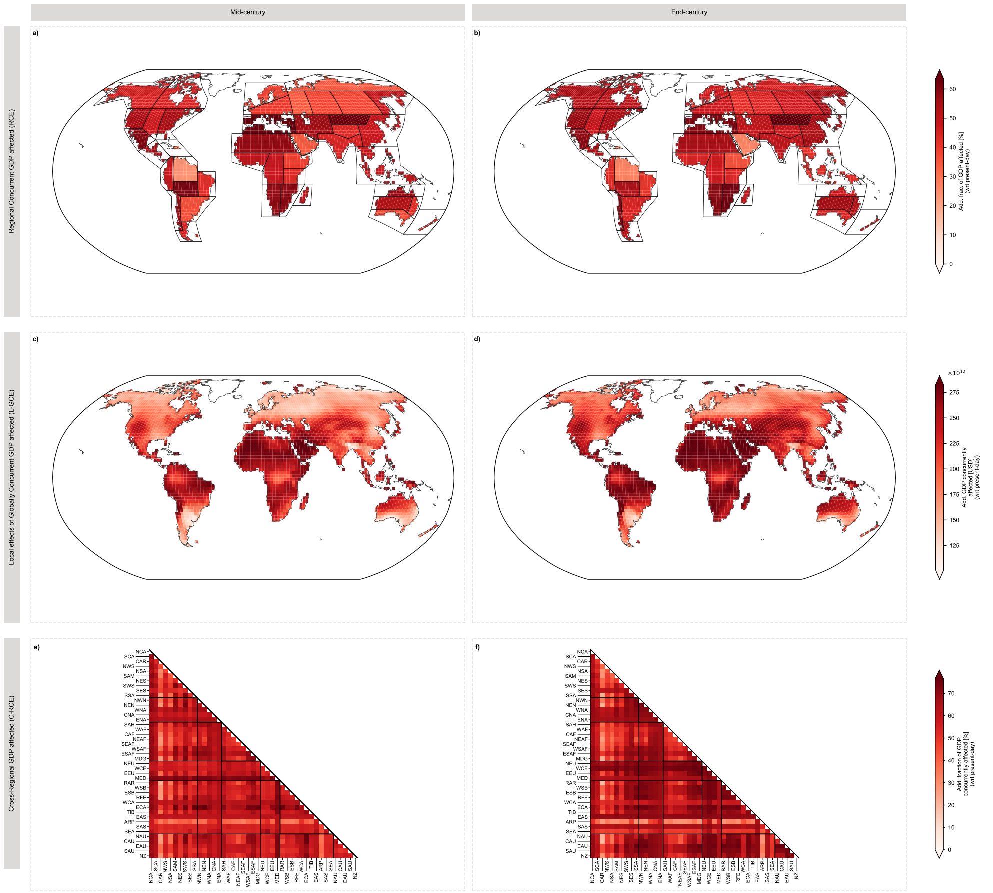

Supplementary Figure 6: Exposure of gross domestic product (GDP) to spatially compounding heatwaves under the Shared Socioeconomic Pathway 2 (SSP2) narrative combined with a radiative forcing level of 4.5 W m^{-2} (SSP2-4.5), shown relative to present-day climate conditions (2001–2020). **Regional Concurrent GDP affected (RCE):** Regional distribution of additional GDP affected by heatwaves for (a) mid-century (2041-2060) and (b) end-century (2081-2100) horizons. **Local Effects of Global Concurrent GDP affected (L-GCE):** Regional distribution of additional GDP affected by heatwaves for (c) mid-century and (d) end-century horizons. **Cross-Regional Concurrent GDP affected (C-RCE):** Additional C-RCE affected by heatwaves for (e) mid-century and (f) end-century horizons.

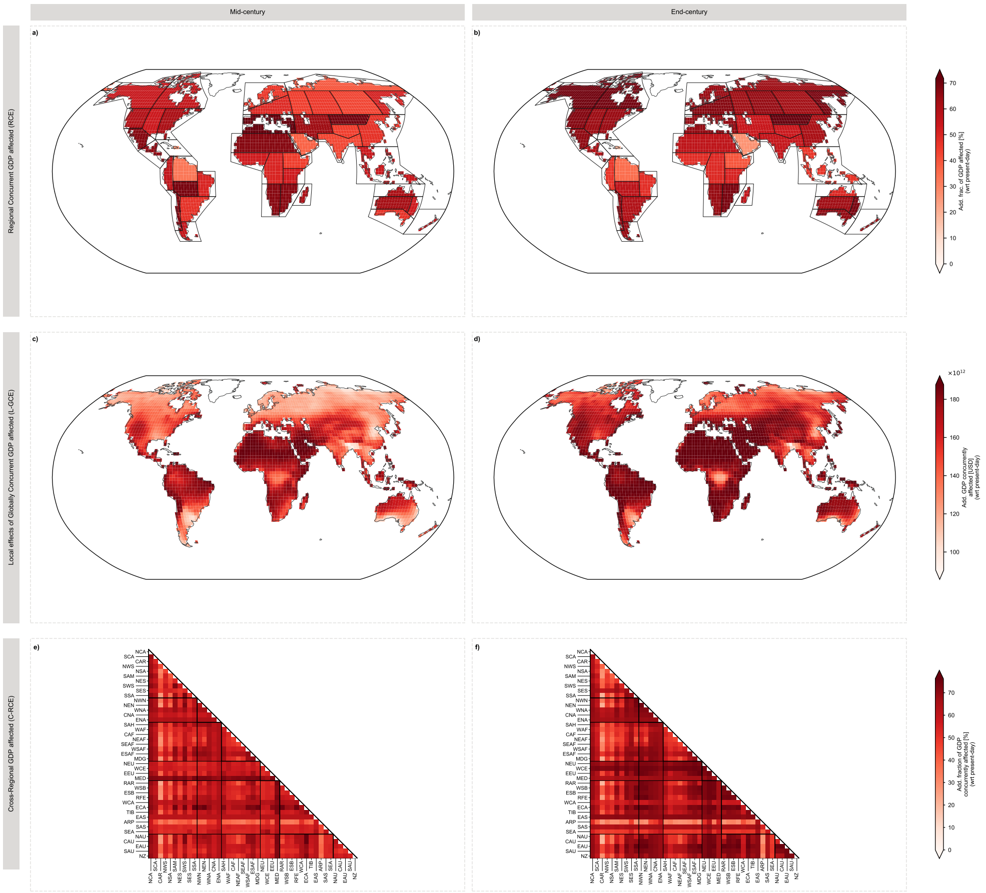

Supplementary Figure 7: Exposure of gross domestic product (GDP) to spatially compounding heatwaves under the Shared Socioeconomic Pathway 3 (SSP3) narrative combined with a radiative forcing level of 7.0 W m^{-2} (SSP3-7.0), shown relative to present-day climate conditions (2001–2020). **Regional Concurrent GDP affected (RCE):** Regional distribution of additional GDP affected by heatwaves for (a) mid-century (2041-2060) and (b) end-century (2081-2100) horizons. **Local Effects of Global Concurrent GDP affected (L-GCE):** Regional distribution of additional GDP affected by heatwaves for (c) mid-century and (d) end-century horizons. **Cross-Regional Concurrent GDP affected (C-RCE):** Additional C-RCE affected by heatwaves for (e) mid-century and (f) end-century horizons.

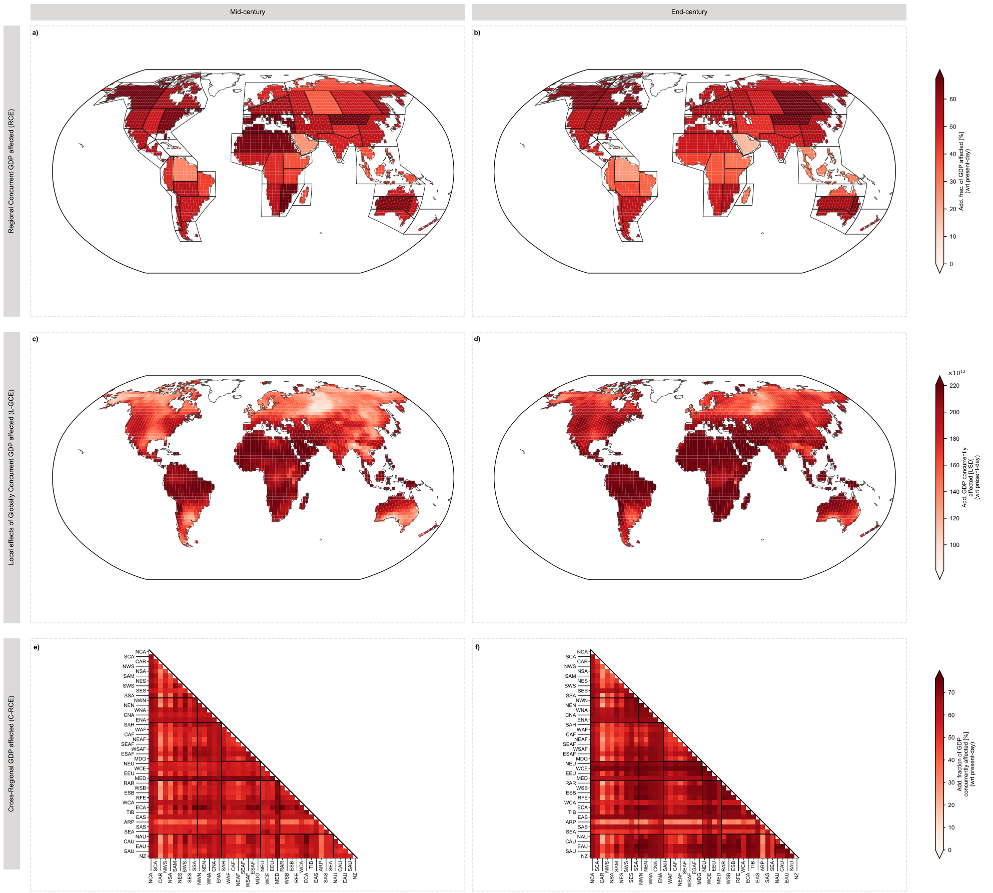

Supplementary Figure 8: Exposure of gross domestic product (GDP) to spatially compounding heatwaves under the Shared Socioeconomic Pathway 4 (SSP4) narrative combined with a radiative forcing level of 6.0 W m^{-2} (SSP4-6.0), shown relative to present-day climate conditions (2001–2020). **Regional Concurrent GDP affected (RCE):** Regional distribution of additional GDP affected by heatwaves for (a) mid-century (2041-2060) and (b) end-century (2081-2100) horizons. **Local Effects of Global Concurrent GDP affected (L-GCE):** Regional distribution of additional GDP affected by heatwaves for (c) mid-century and (d) end-century horizons. **Cross-Regional Concurrent GDP affected (C-RCE):** Additional C-RCE affected by heatwaves for (e) mid-century and (f) end-century horizons.

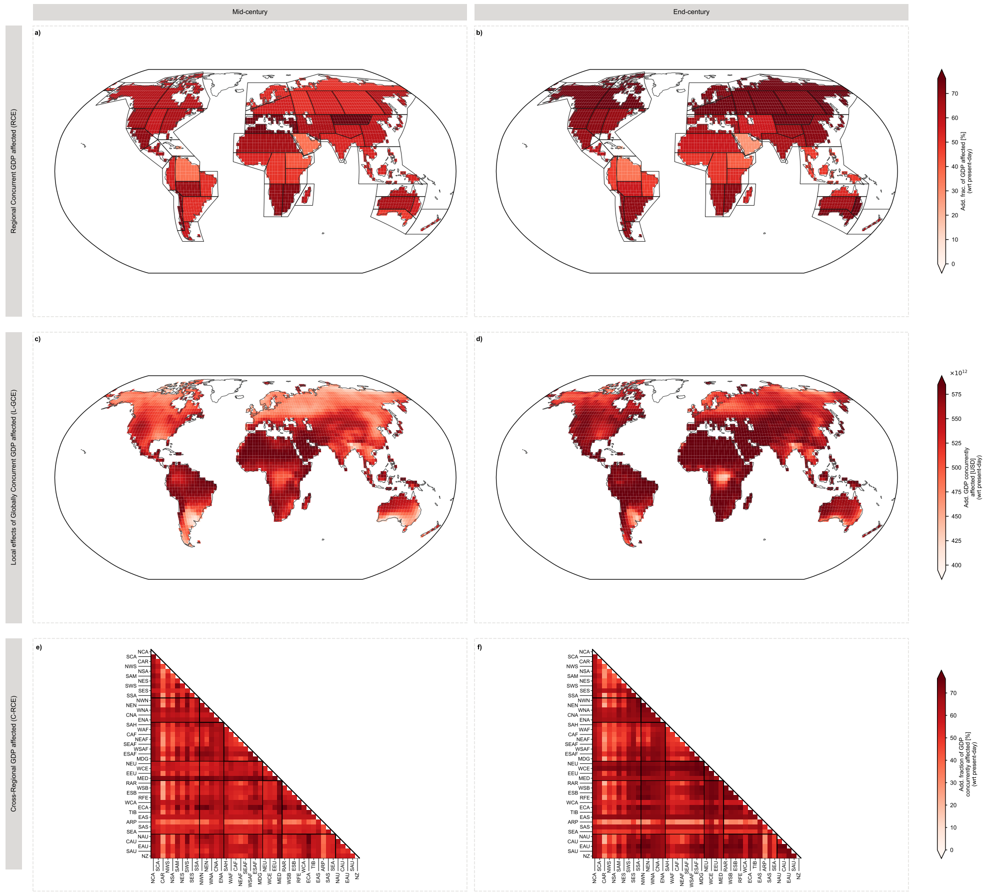

Supplementary Figure 9: Exposure of gross domestic product (GDP) to spatially compounding heatwaves under the Shared Socioeconomic Pathway 5 (SSP5) narrative combined with a radiative forcing level of 8.5 W m^{-2} (SSP5-8.5), shown relative to present-day climate conditions (2001–2020). **Regional Concurrent GDP affected (RCE):** Regional distribution of additional GDP affected by heatwaves for (a) mid-century (2041-2060) and (b) end-century (2081-2100) horizons. **Local Effects of Global Concurrent GDP affected (L-GCE):** Regional distribution of additional GDP affected by heatwaves for (c) mid-century and (d) end-century horizons. **Cross-Regional Concurrent GDP affected (C-RCE):** Additional C-RCE affected by heatwaves for (e) mid-century and (f) end-century horizons.

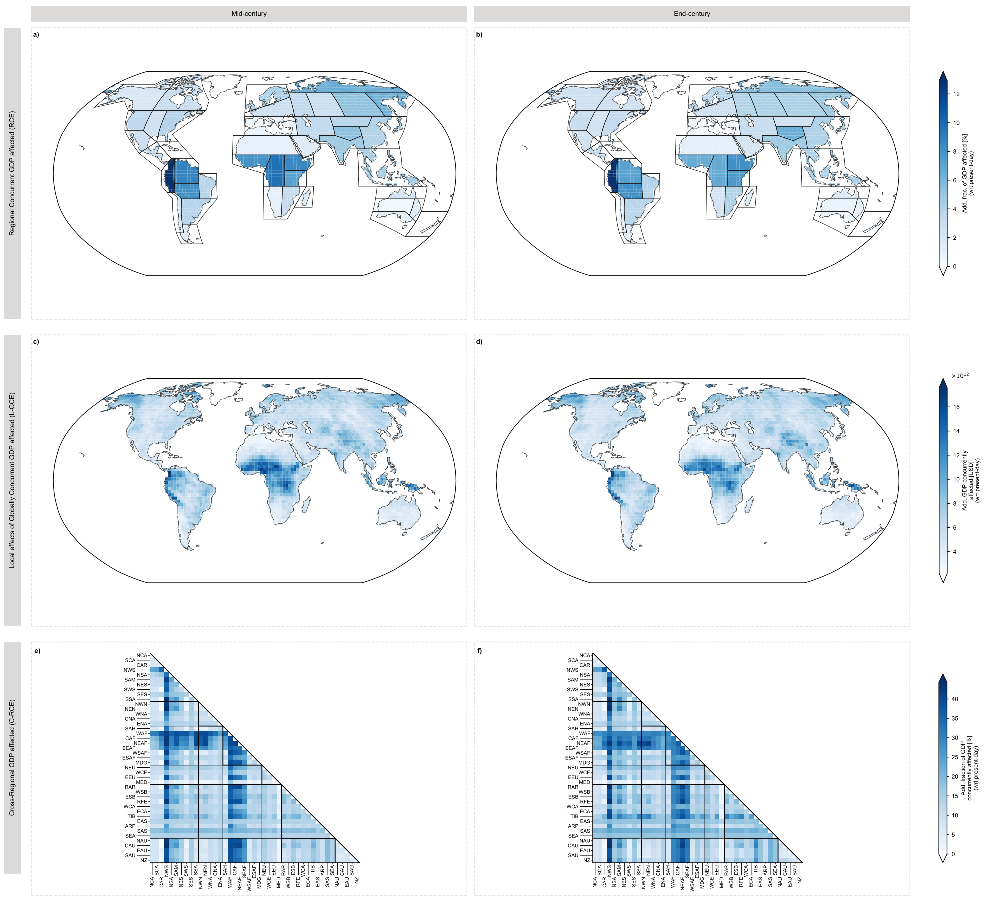

Supplementary Figure 10: Exposure of gross domestic product (GDP) to spatially compounding heavy precipitation under the Shared Socioeconomic Pathway 1 (SSP1) narrative combined with a radiative forcing level of 2.6 W m^{-2} (SSP1-2.6), shown relative to present-day climate conditions (2001–2020). **Regional Concurrent GDP affected (RCE):** Regional distribution of additional GDP affected by heavy precipitation for (a) mid-century (2041-2060) and (b) end-century (2081-2100) horizons. **Local Effects of Global Concurrent GDP affected (L-GCE):** Regional distribution of additional GDP affected by heavy precipitation for (c) mid-century and (d) end-century horizons. **Cross-Regional Concurrent GDP affected (C-RCE):** Additional C-RCE affected by heavy precipitation for (e) mid-century and (f) end-century horizons.

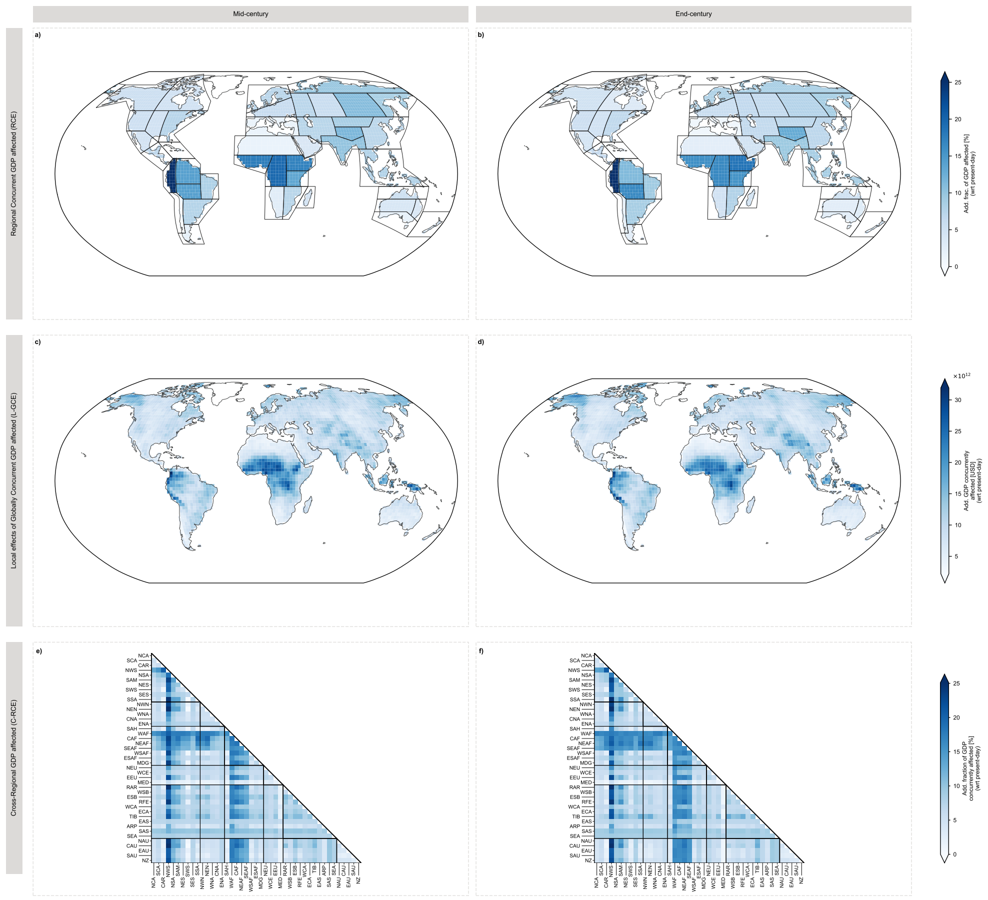

Supplementary Figure 11: Exposure of gross domestic product (GDP) to spatially compounding heavy precipitation under the Shared Socioeconomic Pathway 2 (SSP2) narrative combined with a radiative forcing level of 4.5 W m^{-2} (SSP2-4.5), shown relative to present-day climate conditions (2001–2020). **Regional Concurrent GDP affected (RCE):** Regional distribution of additional GDP affected by heavy precipitation for (a) mid-century (2041-2060) and (b) end-century (2081-2100) horizons. **Local Effects of Global Concurrent GDP affected (L-GCE):** Regional distribution of additional GDP affected by heavy precipitation for (c) mid-century and (d) end-century horizons. **Cross-Regional Concurrent GDP affected (C-RCE):** Additional C-RCE affected by heavy precipitation for (e) mid-century and (f) end-century horizons.

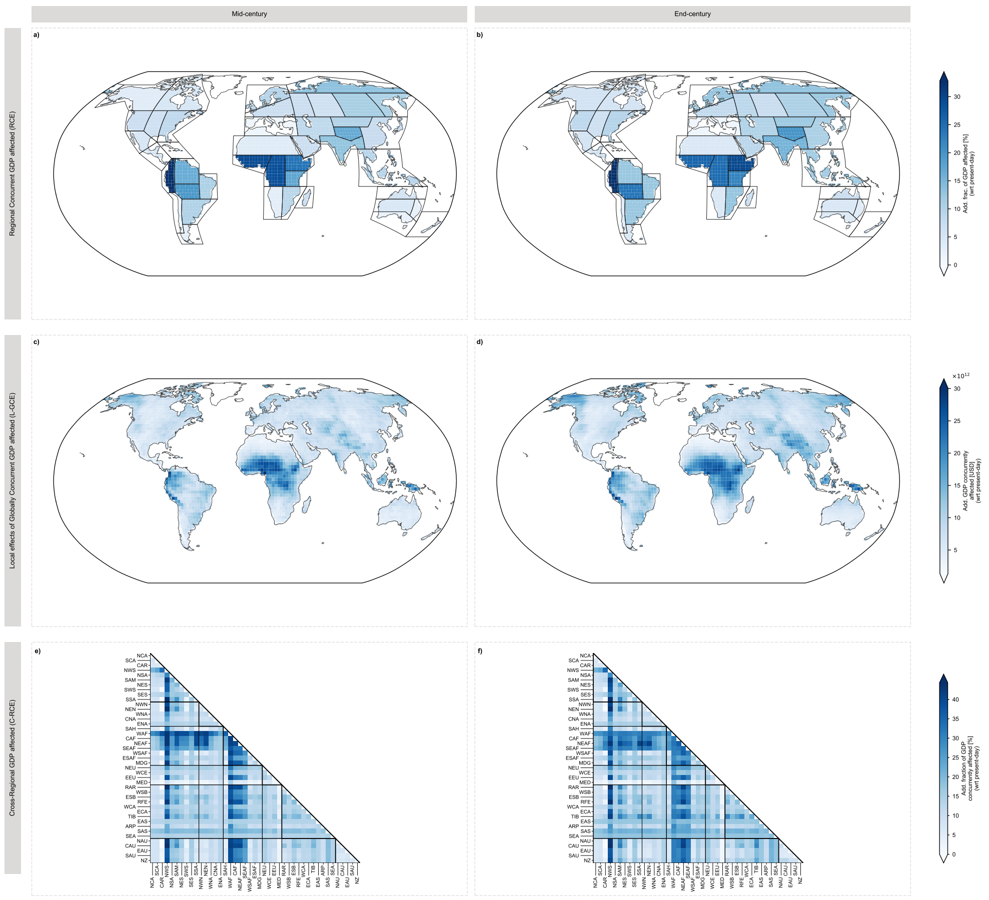

Supplementary Figure 12: Exposure of gross domestic product (GDP) to spatially compounding heavy precipitation under the Shared Socioeconomic Pathway 3 (SSP3) narrative combined with a radiative forcing level of 7.0 W m^{-2} (SSP3-7.0), shown relative to present-day climate conditions (2001–2020). **Regional Concurrent GDP affected (RCE):** Regional distribution of additional GDP affected by heavy precipitation for (a) mid-century (2041-2060) and (b) end-century (2081-2100) horizons. **Local Effects of Global Concurrent GDP affected (L-GCE):** Regional distribution of additional GDP affected by heavy precipitation for (c) mid-century and (d) end-century horizons. **Cross-Regional Concurrent GDP affected (C-RCE):** Additional C-RCE affected by heavy precipitation for (e) mid-century and (f) end-century horizons.

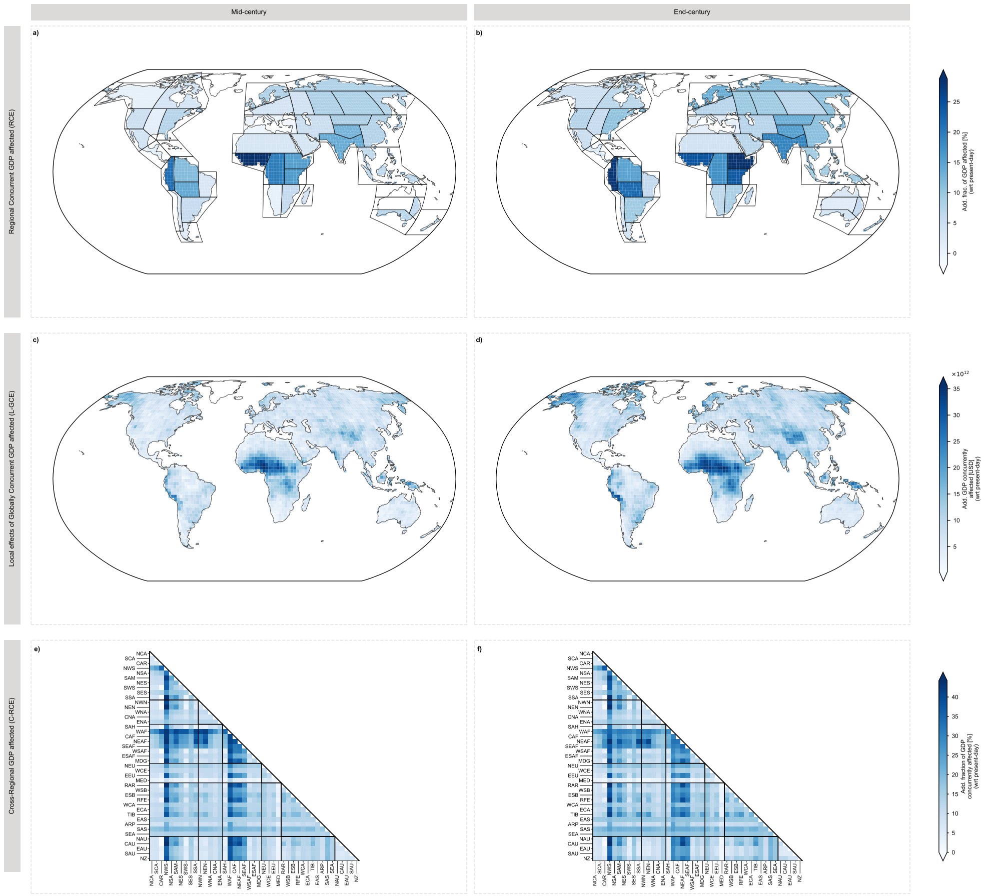

Supplementary Figure 13: Exposure of gross domestic product (GDP) to spatially compounding heavy precipitation under the Shared Socioeconomic Pathway 4 (SSP4) narrative combined with a radiative forcing level of 6.0 W m^{-2} (SSP4-6.0), shown relative to present-day climate conditions (2001–2020). **Regional Concurrent GDP affected (RCE):** Regional distribution of additional GDP affected by heavy precipitation for (a) mid-century (2041-2060) and (b) end-century (2081-2100) horizons. **Local Effects of Global Concurrent GDP affected (L-GCE):** Regional distribution of additional GDP affected by heavy precipitation for (c) mid-century and (d) end-century horizons. **Cross-Regional Concurrent GDP affected (C-RCE):** Additional C-RCE affected by heavy precipitation for (e) mid-century and (f) end-century horizons.

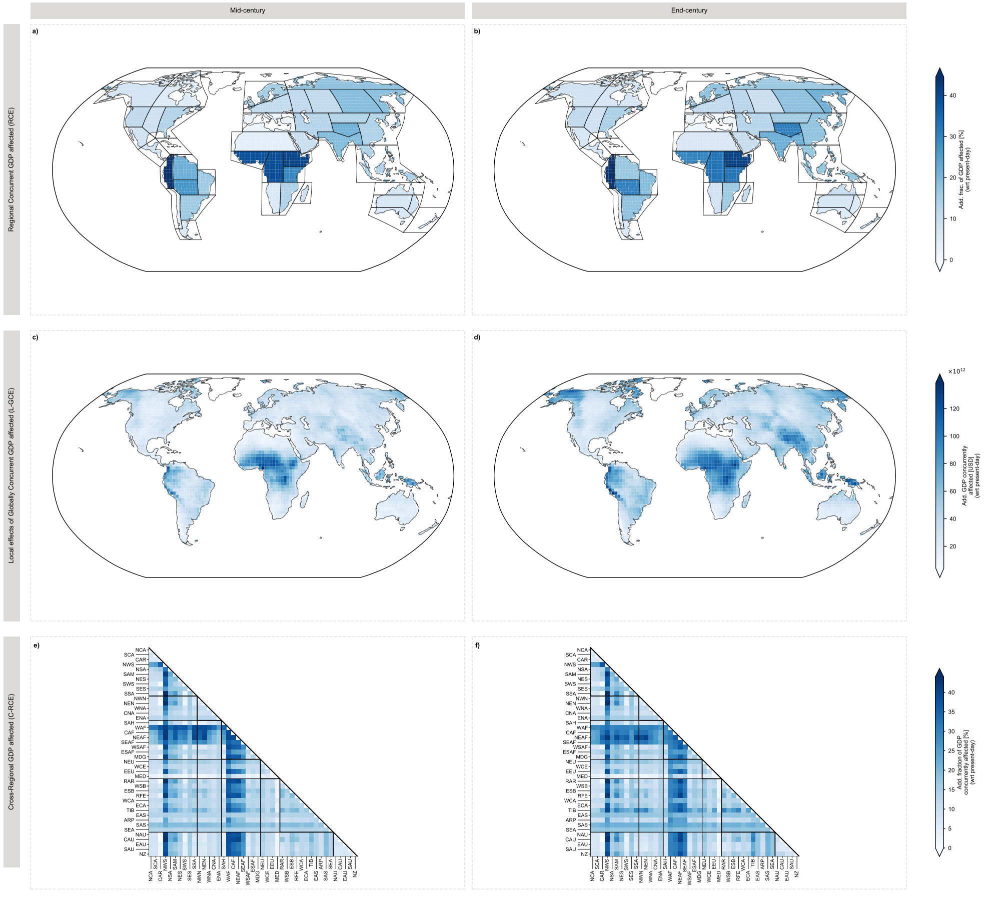

Supplementary Figure 14: Exposure of gross domestic product (GDP) to spatially compounding heavy precipitation under the Shared Socioeconomic Pathway 5 (SSP5) narrative combined with a radiative forcing level of 8.5 W m^{-2} (SSP5-8.5), shown relative to present-day climate conditions (2001–2020). **Regional Concurrent GDP affected (RCE):** Regional distribution of additional GDP affected by heavy precipitation for (a) mid-century (2041-2060) and (b) end-century (2081-2100) horizons. **Local Effects of Global Concurrent GDP affected (L-GCE):** Regional distribution of additional GDP affected by heavy precipitation for (c) mid-century and (d) end-century horizons. **Cross-Regional Concurrent GDP affected (C-RCE):** Additional C-RCE affected by heavy precipitation for (e) mid-century and (f) end-century horizons.

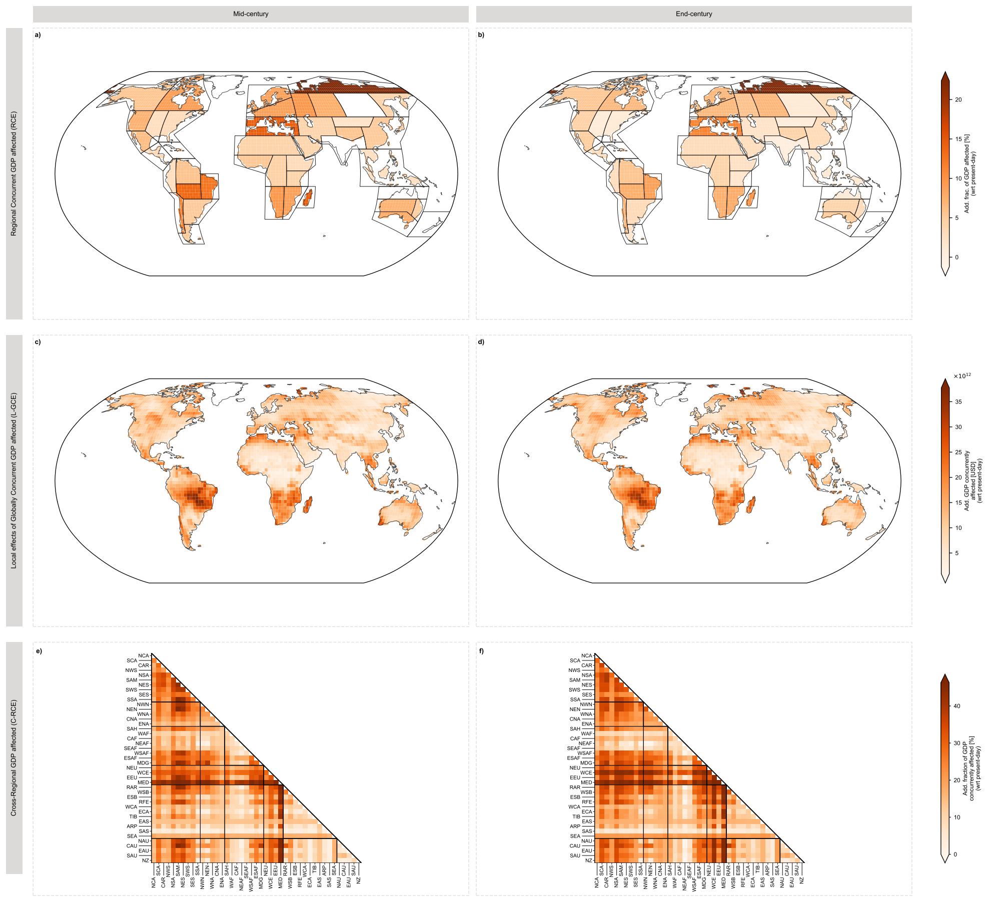

Supplementary Figure 15: Exposure of gross domestic product (GDP) to spatially compounding soil moisture droughts under the Shared Socioeconomic Pathway 1 (SSP1) narrative combined with a radiative forcing level of 2.6 W m^{-2} (SSP1-2.6), shown relative to present-day climate conditions (2001–2020). **Regional Concurrent GDP affected (RCE):** Regional distribution of additional GDP affected by soil moisture droughts for (a) mid-century (2041–2060) and (b) end-century (2081–2100) horizons. **Local Effects of Global Concurrent GDP affected (L-GCE):** Regional distribution of additional GDP affected by soil moisture droughts for (c) mid-century and (d) end-century horizons. **Cross-Regional Concurrent GDP affected (C-RCE):** Additional C-RCE affected by soil moisture droughts for (e) mid-century and (f) end-century horizons.

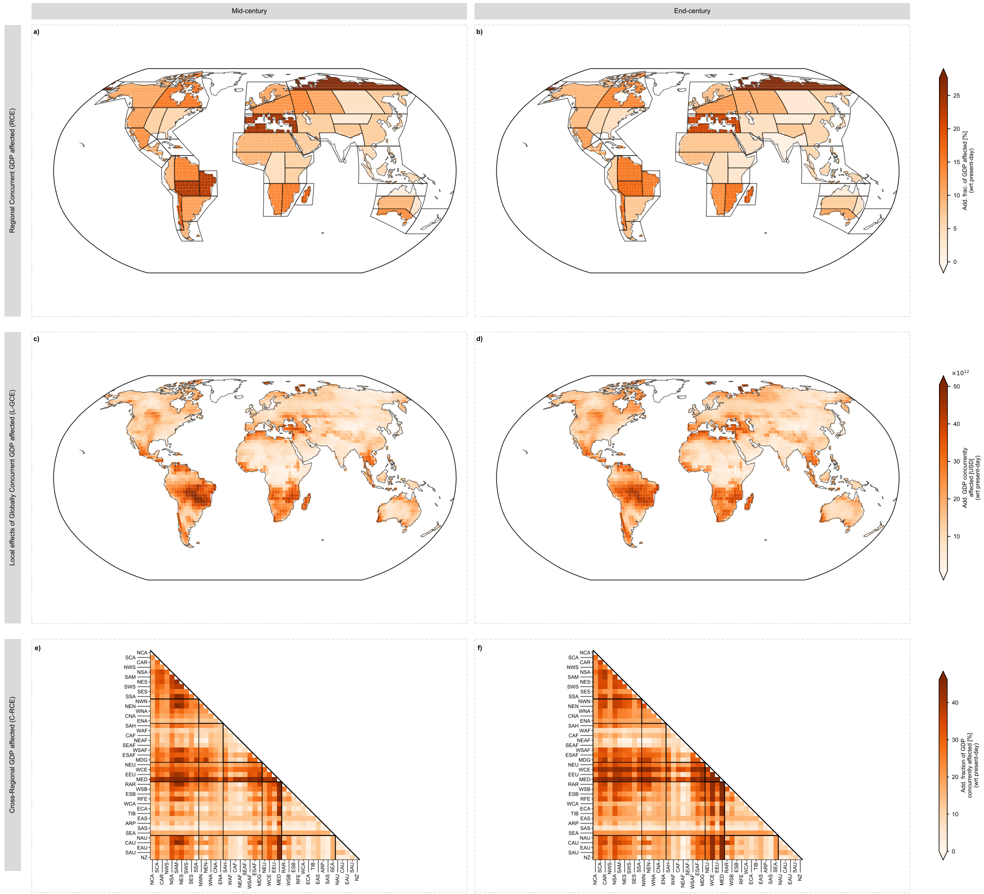

Supplementary Figure 16: Exposure of gross domestic product (GDP) to spatially compounding soil moisture droughts under the Shared Socioeconomic Pathway 2 (SSP2) narrative combined with a radiative forcing level of 4.5 W m^{-2} (SSP2-4.5), shown relative to present-day climate conditions (2001–2020). **Regional Concurrent GDP affected (RCE):** Regional distribution of additional GDP affected by soil moisture droughts for (a) mid-century (2041–2060) and (b) end-century (2081–2100) horizons. **Local Effects of Global Concurrent GDP affected (L-GCE):** Regional distribution of additional GDP affected by soil moisture droughts for (c) mid-century and (d) end-century horizons. **Cross-Regional Concurrent GDP affected (C-RCE):** Additional C-RCE affected by soil moisture droughts for (e) mid-century and (f) end-century horizons.

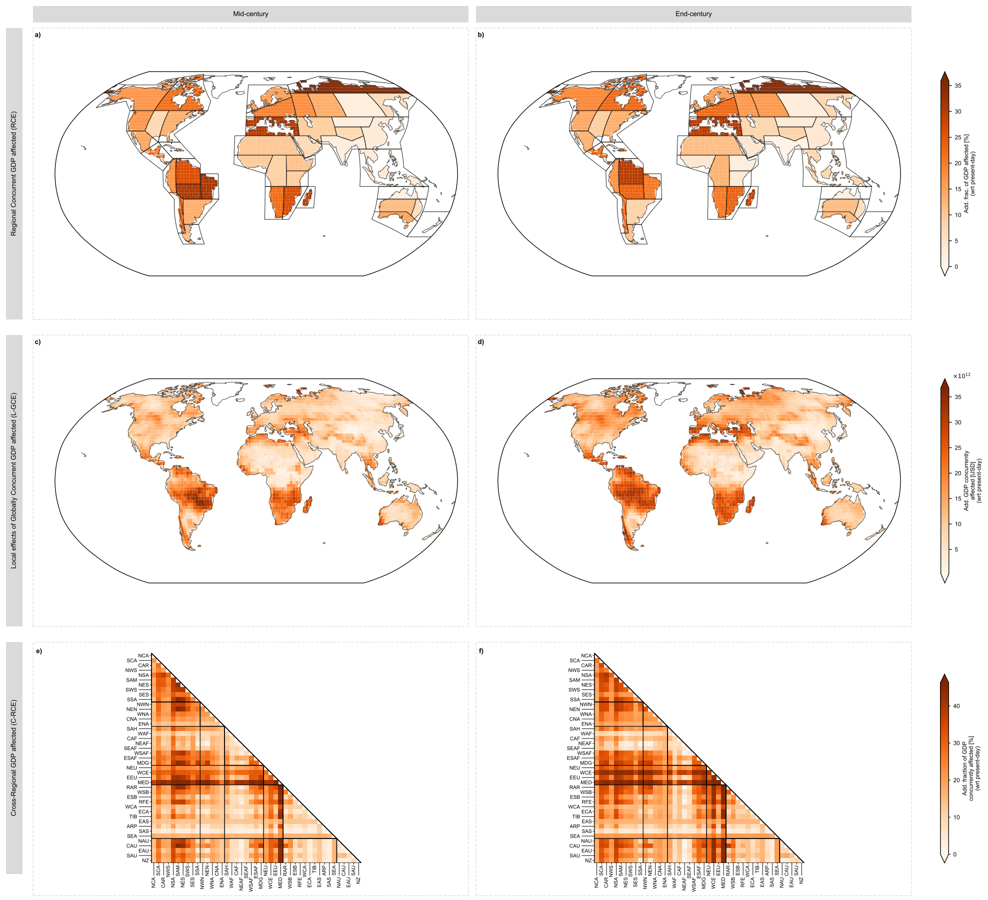

Supplementary Figure 17: Exposure of gross domestic product (GDP) to spatially compounding soil moisture droughts under the Shared Socioeconomic Pathway 3 (SSP3) narrative combined with a radiative forcing level of 7.0 W m^{-2} (SSP3-7.0), shown relative to present-day climate conditions (2001–2020). **Regional Concurrent GDP affected (RCE):** Regional distribution of additional GDP affected by soil moisture droughts for (a) mid-century (2041–2060) and (b) end-century (2081–2100) horizons. **Local Effects of Global Concurrent GDP affected (L-GCE):** Regional distribution of additional GDP affected by soil moisture droughts for (c) mid-century and (d) end-century horizons. **Cross-Regional Concurrent GDP affected (C-RCE):** Additional C-RCE affected by soil moisture droughts for (e) mid-century and (f) end-century horizons.

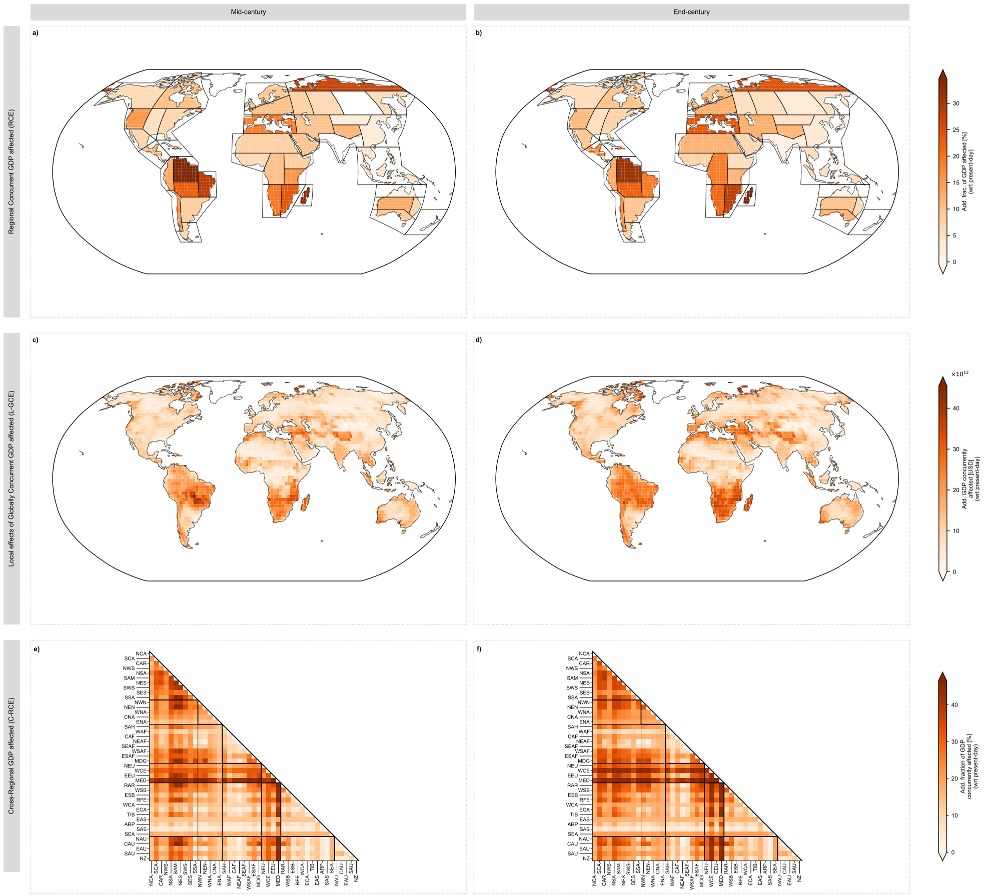

Supplementary Figure 18: Exposure of gross domestic product (GDP) to spatially compounding soil moisture droughts under the Shared Socioeconomic Pathway 4 (SSP4) narrative combined with a radiative forcing level of 6.0 W m^{-2} (SSP4-6.0), shown relative to present-day climate conditions (2001–2020). **Regional Concurrent GDP affected (RCE):** Regional distribution of additional GDP affected by soil moisture droughts for (a) mid-century (2041–2060) and (b) end-century (2081–2100) horizons. **Local Effects of Global Concurrent GDP affected (L-GCE):** Regional distribution of additional GDP affected by soil moisture droughts for (c) mid-century and (d) end-century horizons. **Cross-Regional Concurrent GDP affected (C-RCE):** Additional C-RCE affected by soil moisture droughts for (e) mid-century and (f) end-century horizons.

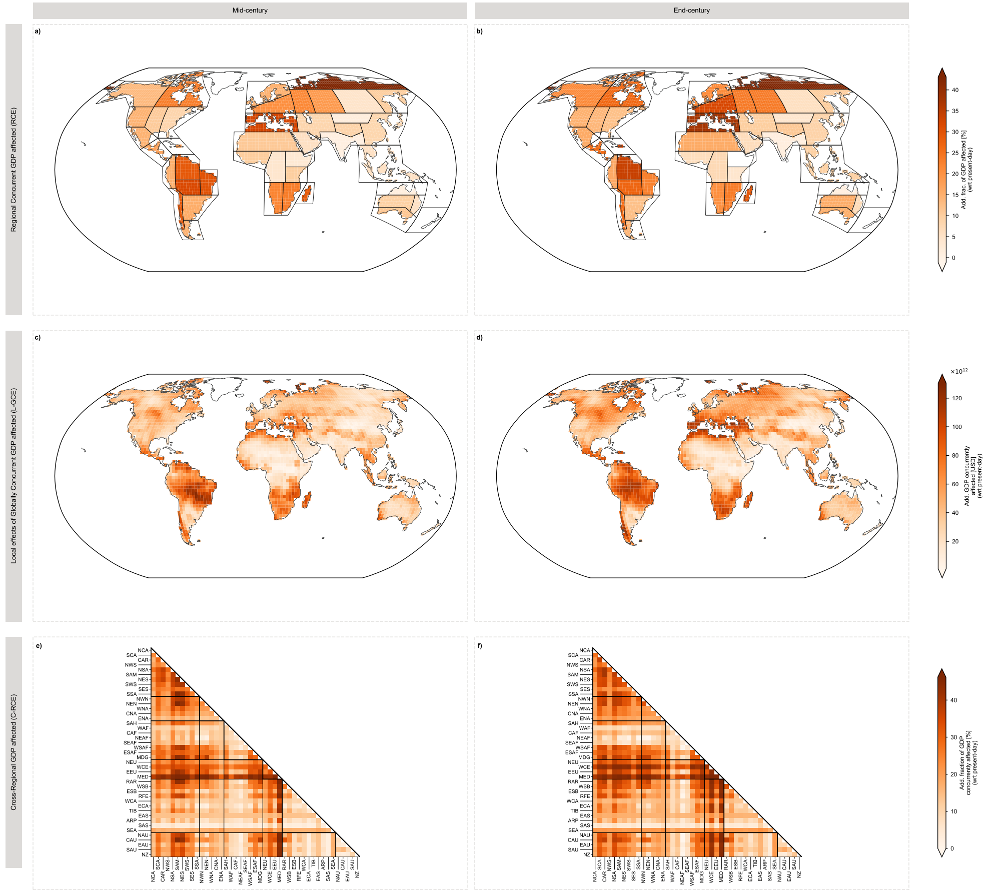

Supplementary Figure 19: Exposure of gross domestic product (GDP) to spatially compounding soil moisture droughts under the Shared Socioeconomic Pathway 5 (SSP5) narrative combined with a radiative forcing level of 8.5 W m^{-2} (SSP5-8.5), shown relative to present-day climate conditions (2001–2020). **Regional Concurrent GDP affected (RCE):** Regional distribution of additional GDP affected by soil moisture droughts for (a) mid-century (2041–2060) and (b) end-century (2081–2100) horizons. **Local Effects of Global Concurrent GDP affected (L-GCE):** Regional distribution of additional GDP affected by soil moisture droughts for (c) mid-century and (d) end-century horizons. **Cross-Regional Concurrent GDP affected (C-RCE):** Additional C-RCE affected by soil moisture droughts for (e) mid-century and (f) end-century horizons.

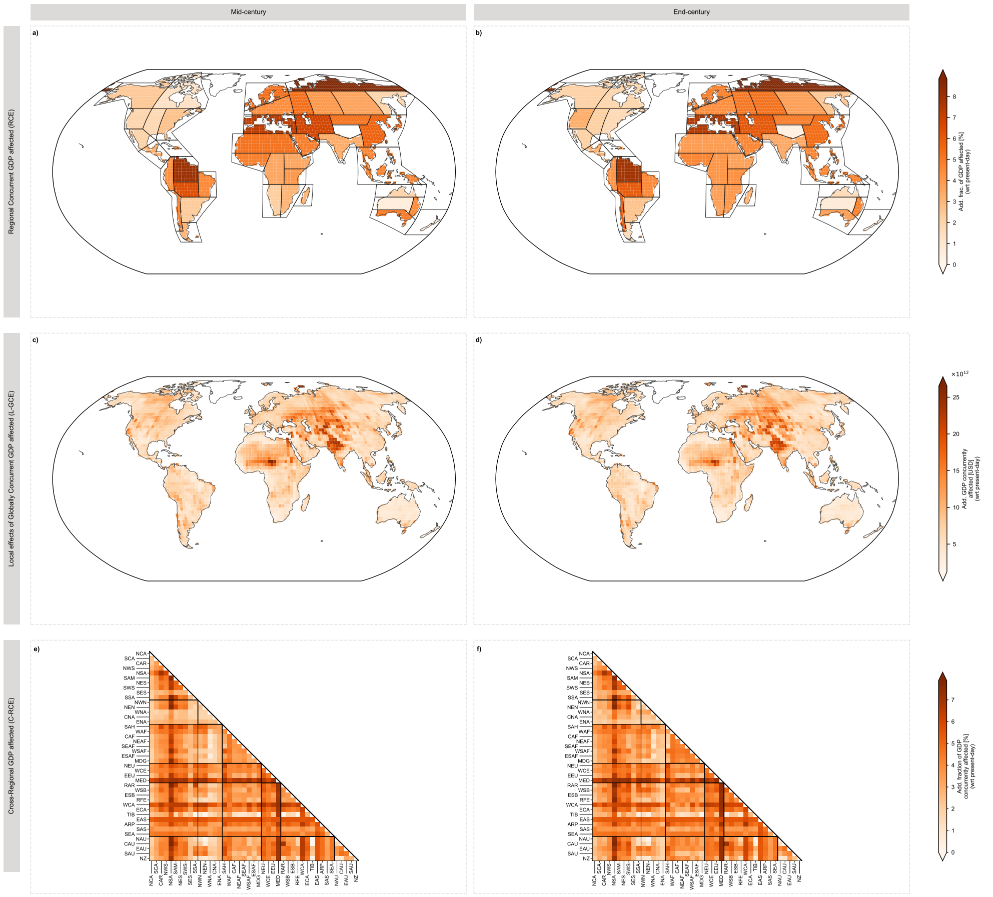

Supplementary Figure 20: Exposure of gross domestic product (GDP) to spatially compounding water scarcity under the Shared Socioeconomic Pathway 1 (SSP1) narrative combined with a radiative forcing level of 2.6 W m^{-2} (SSP1-2.6), shown relative to present-day climate conditions (2001–2020). **Regional Concurrent GDP affected (RCE):** Regional distribution of additional GDP affected by water scarcity for (a) mid-century (2041–2060) and (b) end-century (2081–2100) horizons. **Local Effects of Global Concurrent GDP affected (L-GCE):** Regional distribution of additional GDP affected by water scarcity for (c) mid-century and (d) end-century horizons. **Cross-Regional Concurrent GDP affected (C-RCE):** Additional C-RCE affected by water scarcity for (e) mid-century and (f) end-century horizons.

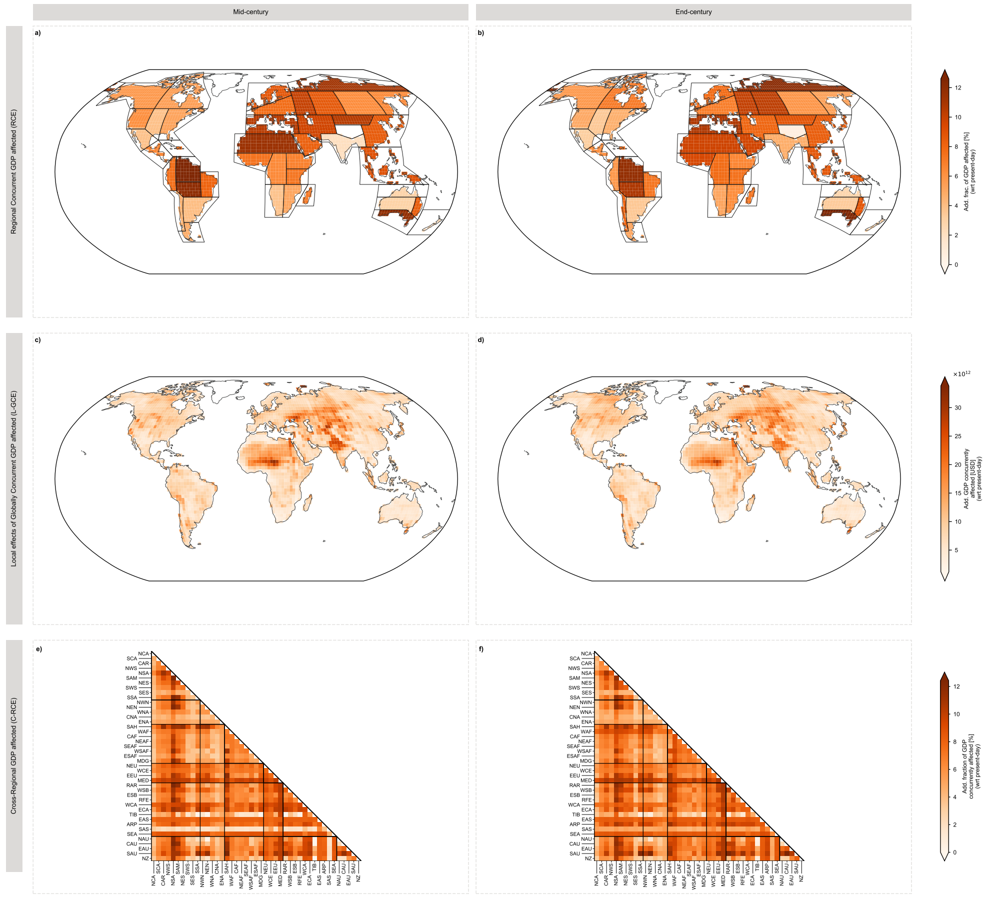

Supplementary Figure 21: Exposure of gross domestic product (GDP) to spatially compounding water scarcity under the Shared Socioeconomic Pathway 2 (SSP2) narrative combined with a radiative forcing level of 4.5 W m^{-2} (SSP2-4.5), shown relative to present-day climate conditions (2001–2020). **Regional Concurrent GDP affected (RCE):** Regional distribution of additional GDP affected by water scarcity for (a) mid-century (2041–2060) and (b) end-century (2081–2100) horizons. **Local Effects of Global Concurrent GDP affected (L-GCE):** Regional distribution of additional GDP affected by water scarcity for (c) mid-century and (d) end-century horizons. **Cross-Regional Concurrent GDP affected (C-RCE):** Additional C-RCE affected by water scarcity for (e) mid-century and (f) end-century horizons.

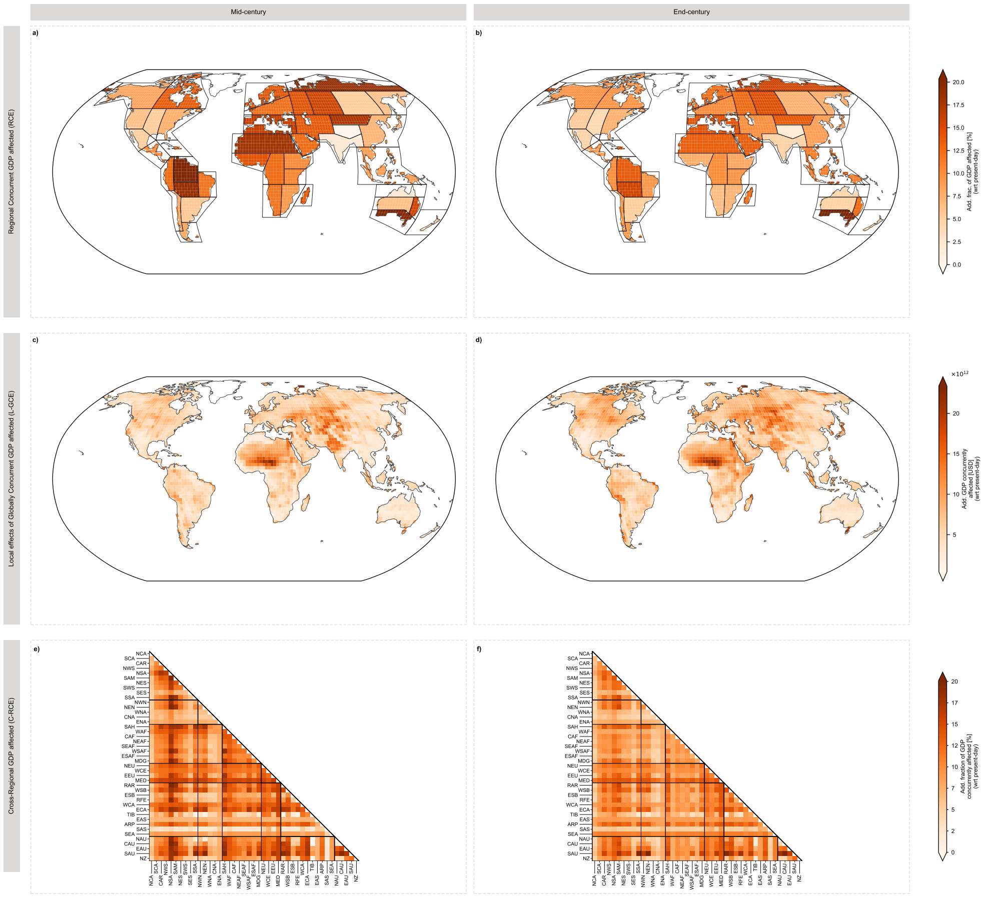

Supplementary Figure 22: Exposure of gross domestic product (GDP) to spatially compounding water scarcity under the Shared Socioeconomic Pathway 3 (SSP3) narrative combined with a radiative forcing level of 7.0 W m^{-2} (SSP3-7.0), shown relative to present-day climate conditions (2001–2020). **Regional Concurrent GDP affected (RCE):** Regional distribution of additional GDP affected by water scarcity for (a) mid-century (2041–2060) and (b) end-century (2081–2100) horizons. **Local Effects of Global Concurrent GDP affected (L-GCE):** Regional distribution of additional GDP affected by water scarcity for (c) mid-century and (d) end-century horizons. **Cross-Regional Concurrent GDP affected (C-RCE):** Additional C-RCE affected by water scarcity for (e) mid-century and (f) end-century horizons.

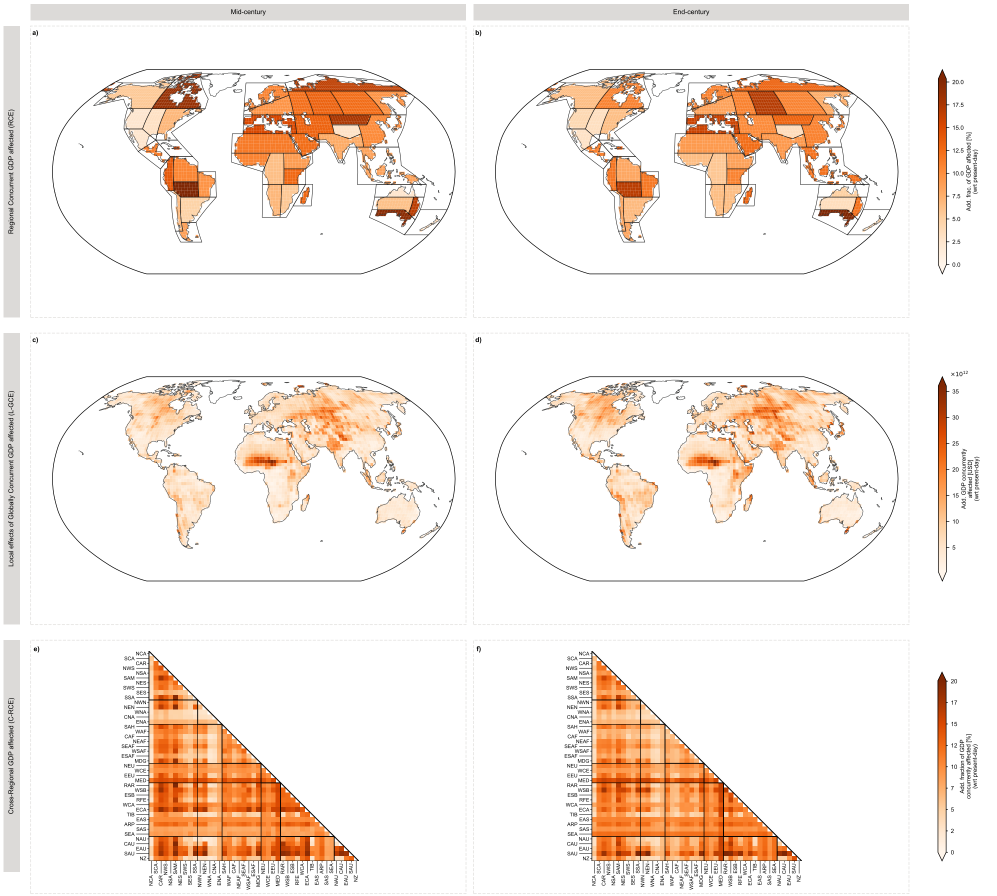

Supplementary Figure 23: Exposure of gross domestic product (GDP) to spatially compounding water scarcity under the Shared Socioeconomic Pathway 4 (SSP4) narrative combined with a radiative forcing level of 6.0 W m^{-2} (SSP4-6.0), shown relative to present-day climate conditions (2001–2020). **Regional Concurrent GDP affected (RCE):** Regional distribution of additional GDP affected by water scarcity for (a) mid-century (2041–2060) and (b) end-century (2081–2100) horizons. **Local Effects of Global Concurrent GDP affected (L-GCE):** Regional distribution of additional GDP affected by water scarcity for (c) mid-century and (d) end-century horizons. **Cross-Regional Concurrent GDP affected (C-RCE):** Additional C-RCE affected by water scarcity for (e) mid-century and (f) end-century horizons.

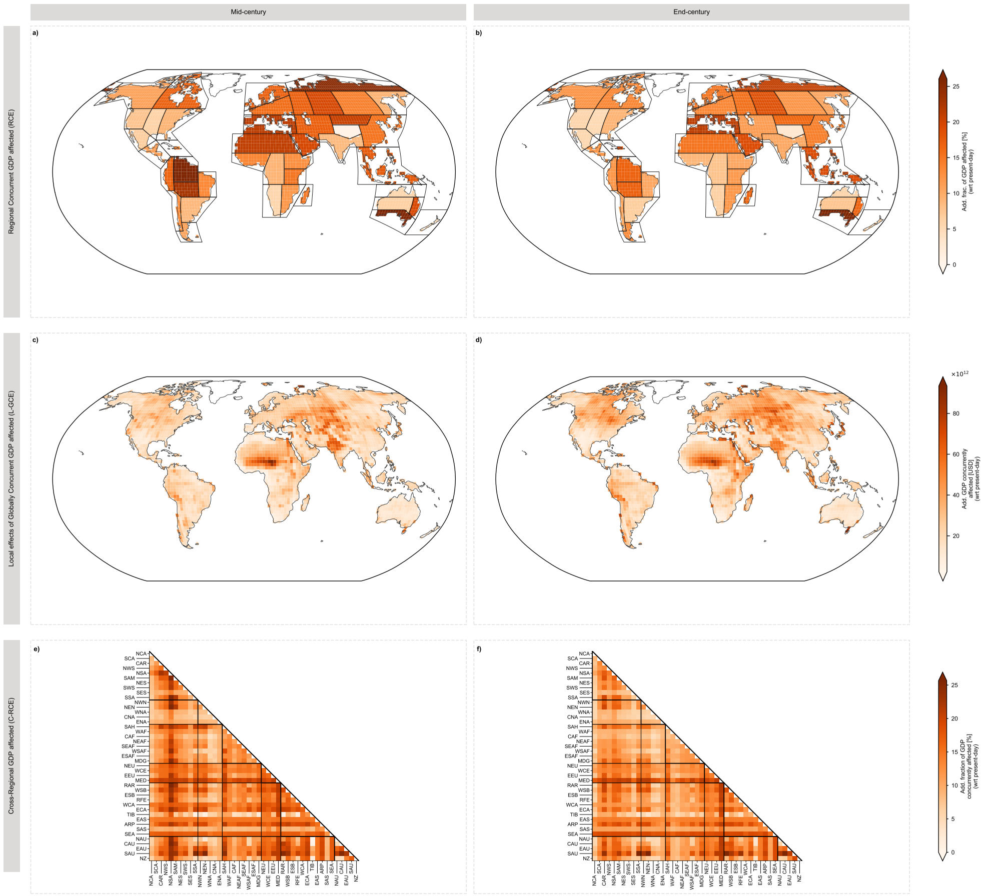

Supplementary Figure 24: Exposure of gross domestic product (GDP) to spatially compounding water scarcity under the Shared Socioeconomic Pathway 5 (SSP5) narrative combined with a radiative forcing level of 8.5 W m^{-2} (SSP5-8.5), shown relative to present-day climate conditions (2001–2020). **Regional Concurrent GDP affected (RCE):** Regional distribution of additional GDP affected by water scarcity for (a) mid-century (2041–2060) and (b) end-century (2081–2100) horizons. **Local Effects of Global Concurrent GDP affected (L-GCE):** Regional distribution of additional GDP affected by water scarcity for (c) mid-century and (d) end-century horizons. **Cross-Regional Concurrent GDP affected (C-RCE):** Additional C-RCE affected by water scarcity for (e) mid-century and (f) end-century horizons.

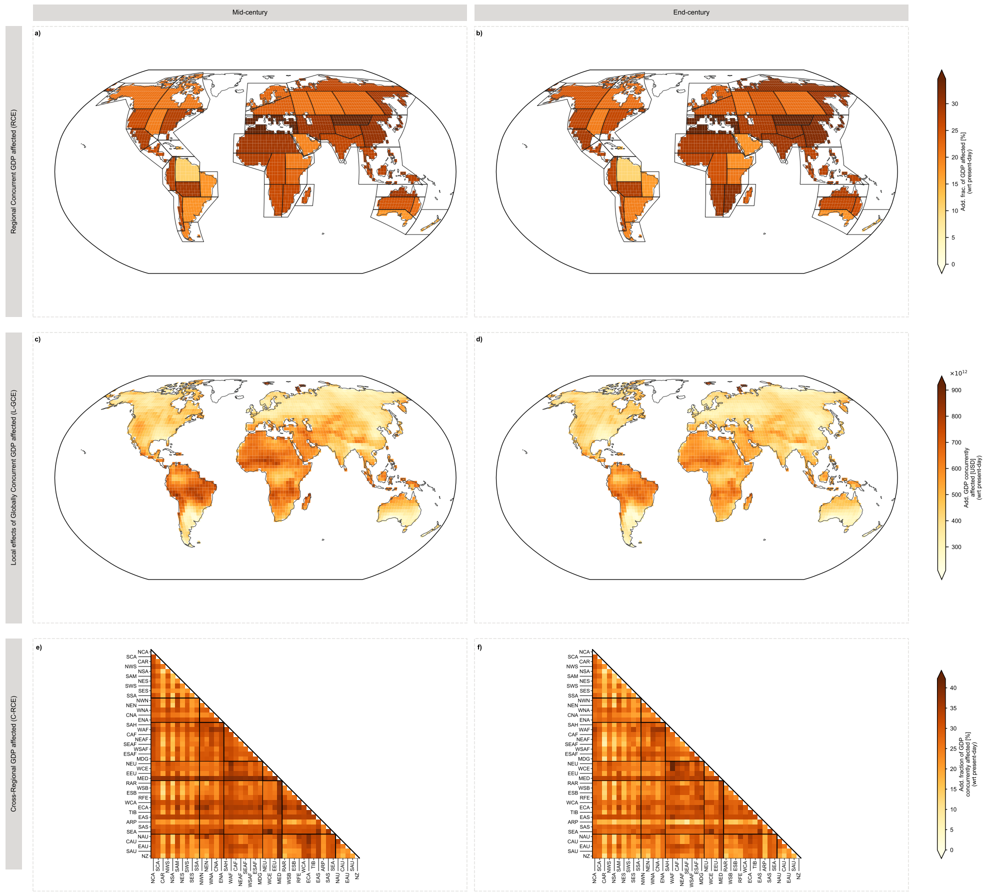

Supplementary Figure 25: Exposure of gross domestic product (GDP) to spatially compounding climate extremes, under the Shared Socioeconomic Pathway 1 (SSP1) narrative combined with a radiative forcing level of 2.6 W m^{-2} (SSP1-2.6), shown relative to present-day climate conditions (2001–2020). Grid cells may be affected by a single extreme-event type—heatwaves, heavy precipitation, water scarcity, or soil-moisture drought—or by any combination of these four hazards. **Concurrent GDP affected (RCE):** Regional distribution of additional GDP affected by at least one extreme-event type under (a) mid-century (2041–2060) and (b) end-century (2081–2100) horizons. **Additional Local Effects of Global Concurrent GDP affected (L-GCE):** Spatial distribution of additional L-GCE for at least one extreme-event type under (c) mid-century and (d) end-century horizons. **Cross-Regional Concurrent GDP affected (C-RCE):** Additional C-RCE for at least one extreme-event type under (e) mid-century and (f) end-century horizons.

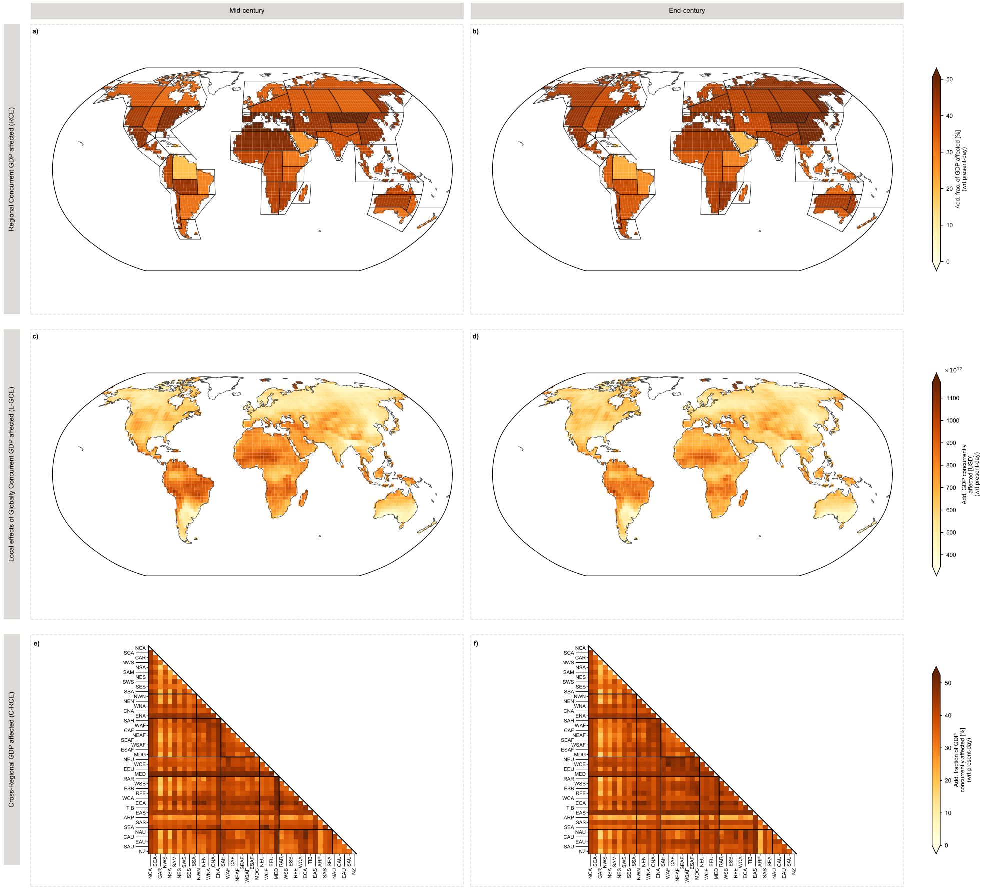

Supplementary Figure 26: Exposure of gross domestic product (GDP) to spatially compounding climate extremes, under the Shared Socioeconomic Pathway 2 (SSP2) narrative combined with a radiative forcing level of 4.5 W m^{-2} (SSP2-4.5), shown relative to present-day climate conditions (2001–2020). Grid cells may be affected by a single extreme-event type—heatwaves, heavy precipitation, water scarcity, or soil-moisture drought—or by any combination of these four hazards. **Concurrent GDP affected (RCE):** Regional distribution of additional GDP affected by at least one extreme-event type under (a) mid-century (2041–2060) and (b) end-century (2081–2100) horizons. **Additional Local Effects of Global Concurrent GDP affected (L-GCE):** Spatial distribution of additional L-GCE for at least one extreme-event type under (c) mid-century and (d) end-century horizons. **Cross-Regional Concurrent GDP affected (C-RCE):** Additional C-RCE for at least one extreme-event type under (e) mid-century and (f) end-century horizons.

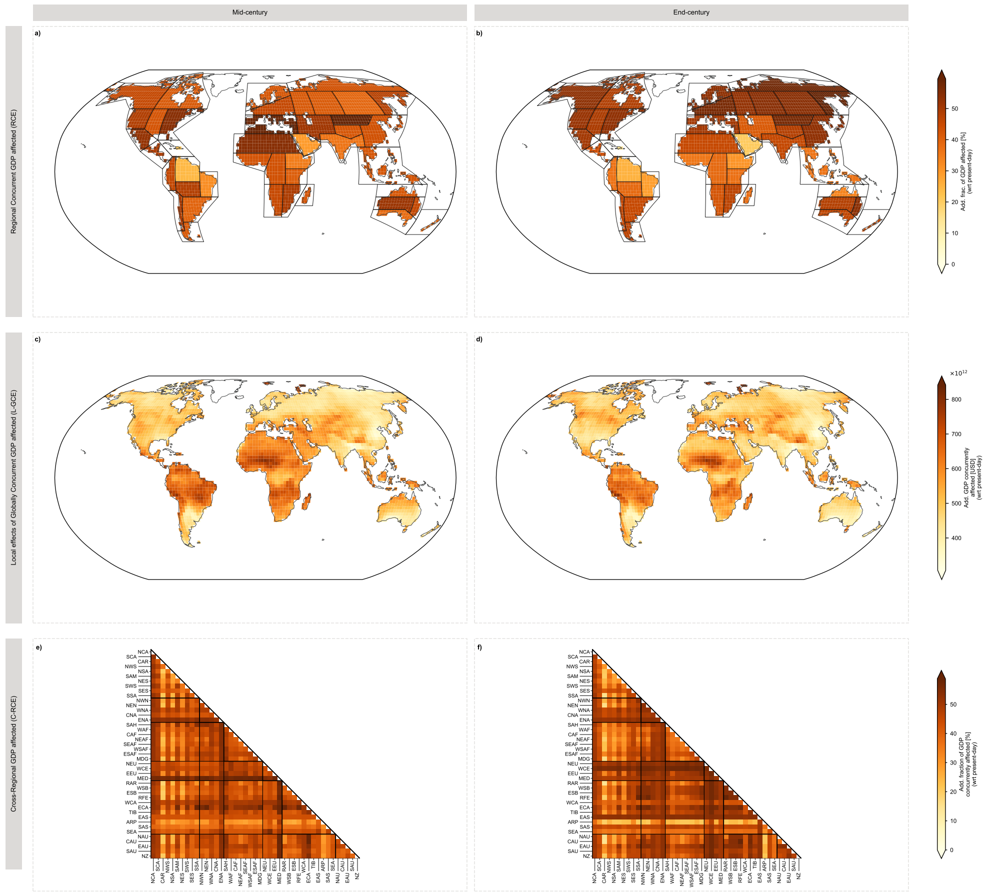

Supplementary Figure 27: Exposure of gross domestic product (GDP) to spatially compounding climate extremes, under the Shared Socioeconomic Pathway 3 (SSP3) narrative combined with a radiative forcing level of 7.0 W m^{-2} (SSP3-7.0), shown relative to present-day climate conditions (2001–2020). Grid cells may be affected by a single extreme-event type—heatwaves, heavy precipitation, water scarcity, or soil-moisture drought—or by any combination of these four hazards. **Concurrent GDP affected (RCE):** Regional distribution of additional GDP affected by at least one extreme-event type under (a) mid-century (2041–2060) and (b) end-century (2081–2100) horizons. **Additional Local Effects of Global Concurrent GDP affected (L-GCE):** Spatial distribution of additional L-GCE for at least one extreme-event type under (c) mid-century and (d) end-century horizons. **Cross-Regional Concurrent GDP affected (C-RCE):** Additional C-RCE for at least one extreme-event type under (e) mid-century and (f) end-century horizons.

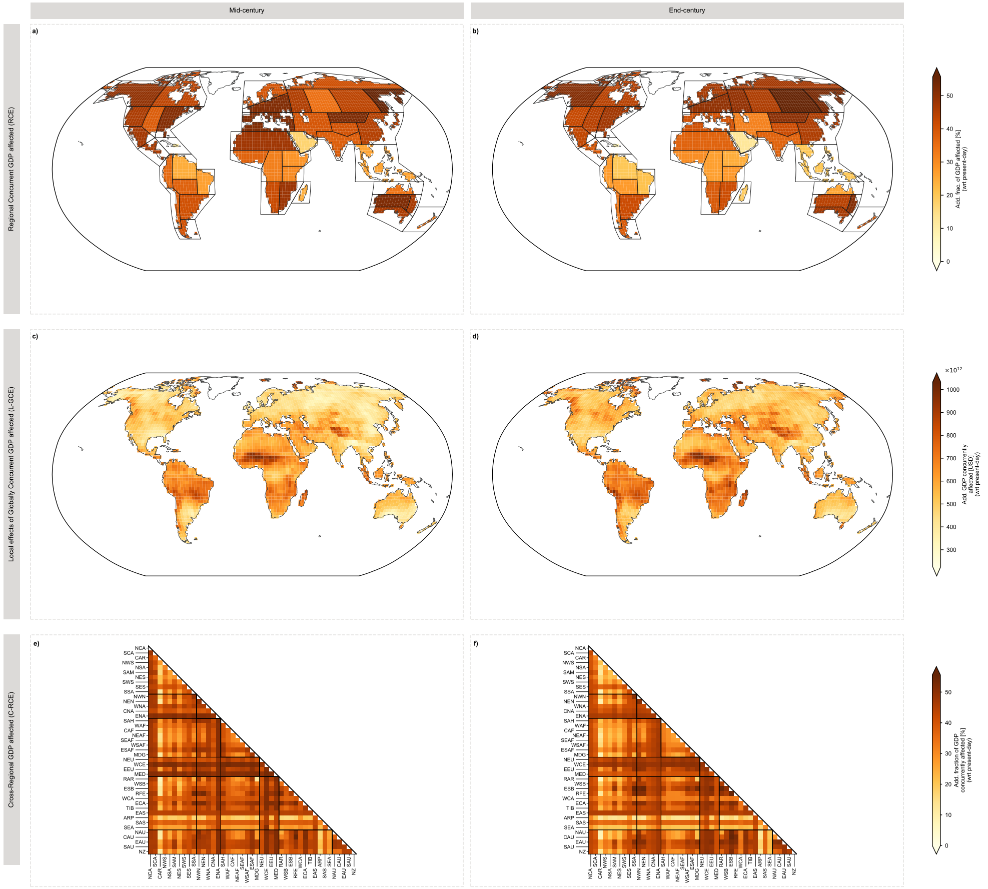

Supplementary Figure 28: Exposure of gross domestic product (GDP) to spatially compounding climate extremes, under the Shared Socioeconomic Pathway 4 (SSP4) narrative combined with a radiative forcing level of 6.0 W m^{-2} (SSP4-6.0), shown relative to present-day climate conditions (2001–2020). Grid cells may be affected by a single extreme-event type—heatwaves, heavy precipitation, water scarcity, or soil-moisture drought—or by any combination of these four hazards. **Concurrent GDP affected (RCE):** Regional distribution of additional GDP affected by at least one extreme-event type under (a) mid-century (2041–2060) and (b) end-century (2081–2100) horizons. **Additional Local Effects of Global Concurrent GDP affected (L-GCE):** Spatial distribution of additional L-GCE for at least one extreme-event type under (c) mid-century and (d) end-century horizons. **Cross-Regional Concurrent GDP affected (C-RCE):** Additional C-RCE for at least one extreme-event type under (e) mid-century and (f) end-century horizons.

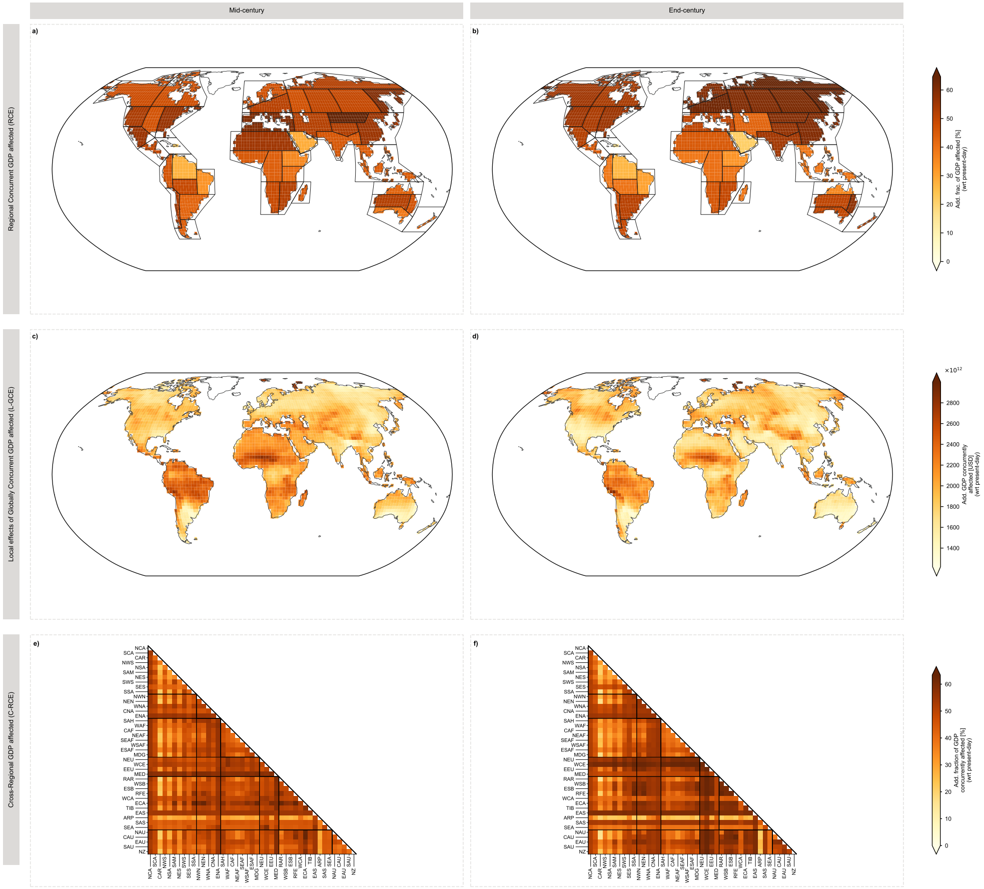

Supplementary Figure 29: Exposure of gross domestic product (GDP) to spatially compounding climate extremes, under the Shared Socioeconomic Pathway 5 (SSP5) narrative combined with a radiative forcing level of 8.5 W m^{-2} (SSP5-8.5), shown relative to present-day climate conditions (2001–2020). Grid cells may be affected by a single extreme-event type—heatwaves, heavy precipitation, water scarcity, or soil-moisture drought—or by any combination of these four hazards. **Concurrent GDP affected (RCE):** Regional distribution of additional GDP affected by at least one extreme-event type under (a) mid-century (2041–2060) and (b) end-century (2081–2100) horizons. **Additional Local Effects of Global Concurrent GDP affected (L-GCE):** Spatial distribution of additional L-GCE for at least one extreme-event type under (c) mid-century and (d) end-century horizons. **Cross-Regional Concurrent GDP affected (C-RCE):** Additional C-RCE for at least one extreme-event type under (e) mid-century and (f) end-century horizons.

Supplementary Table 1: Models and their ensemble members used in this study to assess heatwave occurrences.

**Model**	**Ensemble**	**SSP1-2.6**	**SSP2-4.5**	**SSP3-7.0**	**SSP4-6.0**	**SSP5-8.5**
ACCESS-CM2	r1i1p1f1	x	x	x		x
	r2i1p1f1	x	x	x		x
	r3i1p1f1	x	x	x		x
	r4i1p1f1	x	x	x		x
	r5i1p1f1	x	x	x		x
ACCESS-ESM1-5	r1i1p1f1	x	x	x		x
	r2i1p1f1	x	x	x		x
	r3i1p1f1	x	x	x		x
	r4i1p1f1	x	x	x		x
	r5i1p1f1	x	x	x		x
AWI-CM-1-1-MR	r1i1p1f1	x	x	x		x
	r2i1p1f1			x		
	r3i1p1f1			x		
	r4i1p1f1			x		
	r5i1p1f1			x		
BCC-CSM2-MR	r1i1p1f1	x	x	x		x
CAMS-CSM1-0	r2i1p1f1	x	x	x		x
CMCC-CM2-SR5	r1i1p1f1	x	x			x
CMCC-ESM2	r1i1p1f1	x	x	x		x
CNRM-CM6-1-HR	r1i1p1f2	x				x
CNRM-CM6-1	r1i1p1f2	x	x	x		x
CNRM-ESM2-1	r1i1p1f2	x	x	x	x	x
CanESM5	r1i1p1f1	x	x	x	x	x
	r1i1p2f1	x	x	x		x
	r2i1p1f1	x	x	x	x	x
	r2i1p2f1	x	x	x		x
	r3i1p1f1	x	x	x	x	x
	r4i1p1f1				x	
	r5i1p1f1				x	
EC-Earth3-AerChem	r1i1p1f1			x		
	r3i1p1f1			x		
EC-Earth3-CC	r1i1p1f1		x			x
	r4i1p1f1		x			
	r6i1p1f1		x			
	r7i1p1f1		x			
	r8i1p1f1		x			
EC-Earth3-Veg-LR	r1i1p1f1	x	x	x		x
	r2i1p1f1	x	x	x		x
	r3i1p1f1	x	x	x		x
EC-Earth3-Veg	r12i1p1f1			x		
	r1i1p1f1	x	x	x	x	x
	r2i1p1f1	x	x	x		x

Supplementary Table 1: Models and their ensemble members used in this study to assess heatwave occurrences (continued).

Model	Ensemble	SSP1-2.6	SSP2-4.5	SSP3-7.0	SSP4-6.0	SSP5-8.5
	r3i1p1f1	x	x	x		x
	r4i1p1f1	x	x	x		x
	r5i1p1f1		x			
	r6i1p1f1	x				x
EC-Earth3	r1i1p1f1	x	x	x		x
	r2i1p1f1		x			
	r4i1p1f1	x	x	x		x
	r5i1p1f1	x	x	x		
	r6i1p1f1	x	x	x		x
	r9i1p1f1			x		x
FGOALS-g3	r1i1p1f1	x	x	x	x	x
	r3i1p1f1	x	x	x		x
	r4i1p1f1	x	x	x		x
	r5i1p1f1			x		
GFDL-CM4	r1i1p1f1					x
	r1i1p1f1	x	x	x		x
GISS-E2-1-G	r1i1p1f2	x	x	x	x	x
HadGEM3-GC31-LL	r1i1p1f3	x	x			x
	r2i1p1f3		x			x
	r3i1p1f3		x			x
	r4i1p1f3		x			x
	r5i1p1f3		x			
HadGEM3-GC31-MM	r1i1p1f3	x				x
	r2i1p1f3					x
	r3i1p1f3					x
	r4i1p1f3					x
INM-CM4-8	r1i1p1f1	x	x	x		x
INM-CM5-0	r1i1p1f1	x	x	x		x
	r2i1p1f1			x		
	r3i1p1f1			x		
	r4i1p1f1			x		
	r5i1p1f1			x		
IPSL-CM6A-LR	r1i1p1f1	x	x	x	x	x
	r2i1p1f1	x	x	x	x	x
	r3i1p1f1	x	x	x	x	x
	r4i1p1f1	x	x	x	x	x
	r5i1p1f1		x	x		
	r6i1p1f1	x				x
KACE-1-0-G	r1i1p1f1	x	x	x		x
	r2i1p1f1	x	x	x		x
	r3i1p1f1	x	x	x		x
KIOST-ESM	r1i1p1f1	x	x			x

Supplementary Table 1: Models and their ensemble members used in this study to assess heatwave occurrences (continued).

Model	Ensemble	SSP1-2.6	SSP2-4.5	SSP3-7.0	SSP4-6.0	SSP5-8.5
MIROC-ES2L	r1i1p1f2	x	x	x		x
	r2i1p1f2	x	x	x		x
	r3i1p1f2	x	x	x		x
	r4i1p1f2	x	x	x		x
	r5i1p1f2	x	x	x		x
MIROC6	r1i1p1f1	x	x	x	x	x
	r2i1p1f1	x	x	x		x
	r3i1p1f1	x	x	x		x
	r4i1p1f1	x	x	x		x
	r5i1p1f1	x	x	x		x
MPI-ESM1-2-HR	r1i1p1f1	x	x	x		x
	r2i1p1f1	x	x	x		x
	r3i1p1f1			x		
	r4i1p1f1			x		
	r5i1p1f1			x		
MPI-ESM1-2-LR	r1i1p1f1	x	x	x		x
	r2i1p1f1	x	x	x		x
	r3i1p1f1	x	x	x		x
	r4i1p1f1	x	x	x		x
	r5i1p1f1	x	x	x		x
MRI-ESM2-0	r1i1p1f1	x	x	x	x	x
	r1i2p1f1					x
	r2i1p1f1	x	x	x		x
	r3i1p1f1	x	x	x		x
	r4i1p1f1	x	x	x		x
	r5i1p1f1	x	x	x		
NESM3	r1i1p1f1	x	x			x
NorESM2-LM	r1i1p1f1	x	x	x		x
	r2i1p1f1		x			
	r3i1p1f1		x			
NorESM2-MM	r1i1p1f1	x	x	x		x
	r2i1p1f1		x			
TaiESM1	r1i1p1f1	x	x	x		x
UKESM1-0-LL	r1i1p1f2	x	x	x		x
	r2i1p1f2	x	x	x		x
	r3i1p1f2	x	x	x		x
	r4i1p1f2	x	x	x		x
	r8i1p1f2					x

Supplementary Table 2: Models and their ensemble members used in this study to assess heavy precipitation occurrences.

Model	Ensemble	SSP1-2.6	SSP2-4.5	SSP3-7.0	SSP4-6.0	SSP5-8.5
ACCESS-CM2	r1i1p1f1	x	x	x		x
	r2i1p1f1	x	x	x		x
	r3i1p1f1	x	x	x		x
	r4i1p1f1	x	x	x		x
	r5i1p1f1	x	x	x		x
ACCESS-ESM1-5	r1i1p1f1	x	x	x		x
	r2i1p1f1	x	x	x		x
	r3i1p1f1	x	x	x		x
	r4i1p1f1	x	x	x		x
	r5i1p1f1	x	x	x		x
BCC-CSM2-MR	r1i1p1f1	x	x	x		x
CAMS-CSM1-0	r2i1p1f1	x	x	x		x
CESM2-WACCM	r1i1p1f1		x	x		
	r2i1p1f1		x	x		x
	r3i1p1f1		x	x		x
CESM2	r11i1p1f1	x		x		x
	r1i1p1f1	x	x	x		x
	r2i1p1f1	x	x	x		x
	r3i1p1f1		x	x		
	r4i1p1f1	x	x	x		x
CMCC-CM2-SR5	r1i1p1f1	x	x	x		x
CMCC-ESM2	r1i1p1f1	x	x	x		x
CNRM-CM6-1-HR	r1i1p1f2	x				x
CNRM-CM6-1	r1i1p1f2	x	x	x		x
CNRM-ESM2-1	r1i1p1f2	x	x	x	x	x
CanESM5	r1i1p1f1	x	x	x	x	x
	r1i1p2f1	x	x	x		x
	r2i1p1f1	x	x	x	x	x
	r2i1p2f1	x	x	x		x
	r3i1p1f1	x	x	x	x	x
	r4i1p1f1				x	
	r5i1p1f1				x	
EC-Earth3-AerChem	r1i1p1f1			x		
	r3i1p1f1			x		
EC-Earth3-CC	r1i1p1f1		x	x		x
EC-Earth3-Veg-LR	r1i1p1f1	x	x	x		x
	r2i1p1f1	x	x	x		x
	r3i1p1f1	x	x	x		x
EC-Earth3-Veg	r12i1p1f1			x		
	r1i1p1f1	x	x	x	x	x
	r2i1p1f1	x	x	x		x
	r3i1p1f1	x	x	x		x

Supplementary Table 2: Models and their ensemble members used in this study to assess heavy precipitation occurrences (continued).

Model	Ensemble	SSP1-2.6	SSP2-4.5	SSP3-7.0	SSP4-6.0	SSP5-8.5
	r4i1p1f1	x	x	x		x
	r5i1p1f1		x	x		
	r6i1p1f1	x				x
EC-Earth3	r1i1p1f1	x	x	x		x
	r2i1p1f1		x	x		
	r4i1p1f1	x	x	x		x
	r5i1p1f1	x	x	x		
	r6i1p1f1	x	x	x		x
	r9i1p1f1			x		x
FGOALS-g3	r1i1p1f1	x	x	x	x	x
	r3i1p1f1	x	x	x		x
	r4i1p1f1	x	x	x		x
	r5i1p1f1			x		
GFDL-CM4	r1i1p1f1		x	x		x
GFDL-ESM4	r1i1p1f1	x	x	x		x
HadGEM3-GC31-LL	r1i1p1f3	x	x	x		x
	r2i1p1f3		x	x		x
	r3i1p1f3		x	x		x
	r4i1p1f3		x	x		x
	r5i1p1f3		x	x		
HadGEM3-GC31-MM	r1i1p1f3	x				x
	r2i1p1f3					x
	r3i1p1f3					x
INM-CM4-8	r1i1p1f1	x	x	x		x
INM-CM5-0	r1i1p1f1	x	x	x		x
	r2i1p1f1			x		
	r3i1p1f1			x		
	r4i1p1f1			x		
	r5i1p1f1			x		
IPSL-CM5A2-INCA	r1i1p1f1	x		x		
IPSL-CM6A-LR	r1i1p1f1	x	x	x	x	x
	r2i1p1f1	x	x	x	x	x
	r3i1p1f1	x	x	x	x	x
	r4i1p1f1	x	x	x	x	x
	r5i1p1f1		x	x		
	r6i1p1f1	x				x
KACE-1-0-G	r1i1p1f1	x	x	x		x
	r2i1p1f1	x	x	x		x
	r3i1p1f1	x	x	x		x
KIOST-ESM	r1i1p1f1	x	x	x		x
MIROC-ES2L	r1i1p1f2	x	x	x		x
	r2i1p1f2	x	x	x		x

Supplementary Table 2: Models and their ensemble members used in this study to assess heavy precipitation occurrences (continued).

Model	Ensemble	SSP1-2.6	SSP2-4.5	SSP3-7.0	SSP4-6.0	SSP5-8.5
	r3i1p1f2	x	x	x		x
	r4i1p1f2	x	x	x		x
	r5i1p1f2	x	x	x		x
MIROC6	r1i1p1f1	x	x	x	x	x
	r2i1p1f1	x	x	x		x
	r3i1p1f1	x	x	x		x
	r4i1p1f1	x	x	x		x
	r5i1p1f1	x	x	x		x
MPI-ESM1-2-HR	r1i1p1f1	x	x	x		x
	r2i1p1f1	x	x	x		x
	r3i1p1f1			x		
	r4i1p1f1			x		
	r5i1p1f1			x		
MPI-ESM1-2-LR	r1i1p1f1	x	x	x		x
	r2i1p1f1	x	x	x		x
	r3i1p1f1	x	x	x		x
	r4i1p1f1	x	x	x		x
	r5i1p1f1	x	x	x		x
MRI-ESM2-0	r1i1p1f1	x	x	x	x	x
	r1i2p1f1					x
	r2i1p1f1	x	x	x		x
	r3i1p1f1	x	x	x		x
	r4i1p1f1	x	x	x		x
	r5i1p1f1	x	x	x		
NESM3	r1i1p1f1	x	x			x
NorESM2-LM	r1i1p1f1	x	x	x		x
	r2i1p1f1		x			
	r3i1p1f1		x			
NorESM2-MM	r1i1p1f1	x	x	x		x
	r2i1p1f1		x			
TaiESM1	r1i1p1f1	x	x	x		x
UKESM1-0-LL	r1i1p1f2	x	x	x		x
	r2i1p1f2	x	x	x		x
	r3i1p1f2	x	x	x		x
	r4i1p1f2	x	x	x		x
	r8i1p1f2					x

Supplementary Table 3: Models and their ensemble members used in this study to assess water scarcity occurrences.

Model	Ensemble	SSP1-2.6	SSP2-4.5	SSP3-7.0	SSP4-6.0	SSP5-8.5
ACCESS-CM2	r1i1p1f1	x	x	x		x
	r2i1p1f1	x	x	x		x
	r3i1p1f1	x	x	x		x
	r4i1p1f1	x	x	x		x
	r5i1p1f1	x	x	x		x
ACCESS-ESM1-5	r1i1p1f1	x	x	x		x
	r2i1p1f1	x	x	x		x
	r3i1p1f1	x	x	x		x
	r4i1p1f1	x	x	x		x
	r5i1p1f1	x	x	x		x
BCC-CSM2-MR	r1i1p1f1	x	x	x		x
CESM2-FV2	r1i2p2f1			x		x
CESM2-WACCM	r1i1p1f1	x	x	x		x
	r2i1p1f1		x			x
	r3i1p1f1		x			x
CESM2	r10i1p1f1	x	x			x
	r11i1p1f1	x				x
	r1i1p1f1	x	x	x		x
	r2i1p1f1	x	x	x		x
	r3i1p1f1		x	x		
	r4i1p1f1	x	x	x		x
	r5i1p1f1			x		
CMCC-CM2-SR5	r1i1p1f1	x	x	x		x
CMCC-ESM2	r1i1p1f1	x	x	x		x
CNRM-CM6-1-HR	r1i1p1f2	x	x	x		x
CNRM-CM6-1	r1i1p1f2	x	x	x		x
	r2i1p1f2	x	x	x		x
	r3i1p1f2	x	x	x		x
	r4i1p1f2	x	x	x		x
	r5i1p1f2	x	x	x		x
CNRM-ESM2-1	r1i1p1f2	x	x	x	x	x
	r2i1p1f2	x	x	x	x	x
	r3i1p1f2	x	x	x	x	x
	r4i1p1f2	x	x	x	x	x
	r5i1p1f2	x	x	x	x	x
CanESM5-1	r1i1p1f1	x	x	x	x	x
	r1i1p2f1	x	x	x	x	x
	r2i1p1f1		x	x		x
	r2i1p2f1		x	x		x
	r3i1p1f1		x	x		x
CanESM5-CanOE	r1i1p2f1	x	x	x		x
	r2i1p2f1	x	x	x		x

Supplementary Table 3: Models and their ensemble members used in this study to assess water scarcity occurrences (continued).

Model	Ensemble	SSP1-2.6	SSP2-4.5	SSP3-7.0	SSP4-6.0	SSP5-8.5
	r3i1p2f1	x	x	x		x
CanESM5	r1i1p1f1	x	x	x	x	x
	r1i1p2f1	x	x	x		x
	r2i1p1f1	x	x	x	x	x
	r2i1p2f1	x	x	x		x
	r3i1p1f1	x	x	x	x	x
	r4i1p1f1				x	
	r5i1p1f1				x	
E3SM-1-1-ECA	r1i1p1f1					x
E3SM-1-1	r1i1p1f1					x
E3SM-2-0	r1i1p1f1			x		
EC-Earth3-AerChem	r1i1p1f1			x		
EC-Earth3-CC	r1i1p1f1		x			x
	r4i1p1f1		x			
	r6i1p1f1		x			
	r7i1p1f1		x			
	r8i1p1f1		x			
EC-Earth3-Veg-LR	r1i1p1f1	x	x	x		x
	r2i1p1f1	x	x	x		x
	r3i1p1f1	x	x	x		x
EC-Earth3-Veg	r12i1p1f1			x		
	r1i1p1f1	x	x	x	x	x
	r2i1p1f1	x	x	x		x
	r3i1p1f1	x	x	x		x
	r4i1p1f1	x	x	x		x
	r6i1p1f1	x	x			x
EC-Earth3	r1i1p1f1	x	x	x		x
	r2i1p1f1		x			
	r4i1p1f1	x	x	x		x
	r5i1p1f1	x	x	x		
	r6i1p1f1	x	x	x		x
	r9i1p1f1			x		x
FGOALS-f3-L	r1i1p1f1	x	x	x		x
FGOALS-g3	r1i1p1f1	x	x	x	x	x
	r2i1p1f1	x	x	x		x
	r3i1p1f1	x	x	x		x
	r4i1p1f1	x	x	x		x
	r5i1p1f1			x		
FIO-ESM-2-0	r1i1p1f1	x	x			x
	r2i1p1f1	x	x			x
	r3i1p1f1	x	x			x
GFDL-CM4	r1i1p1f1		x			x

Supplementary Table 3: Models and their ensemble members used in this study to assess water scarcity occurrences (continued).

Model	Ensemble	SSP1-2.6	SSP2-4.5	SSP3-7.0	SSP4-6.0	SSP5-8.5
GFDL-ESM4	r1i1p1f1	x	x	x		x
GISS-E2-1-G	r1i1p1f2	x	x	x	x	x
	r1i1p3f1	x	x	x	x	x
	r1i1p5f1	x	x	x	x	x
	r2i1p1f2	x	x	x	x	x
	r2i1p3f1	x	x			x
	r3i1p1f2				x	
GISS-E2-1-H	r1i1p1f2	x	x	x	x	x
	r1i1p3f1	x	x	x	x	x
	r2i1p1f2	x	x	x		x
	r2i1p3f1	x	x			x
	r3i1p1f2	x	x	x		x
	r4i1p1f2			x		
GISS-E2-2-G	r1i1p3f1	x	x	x		x
	r2i1p3f1	x	x	x		x
	r3i1p3f1	x	x	x		x
	r4i1p3f1	x	x	x		x
	r5i1p3f1	x	x	x		x
IPSL-CM6A-LR	r1i1p1f1	x	x	x	x	x
	r2i1p1f1	x	x	x	x	x
	r3i1p1f1	x	x	x	x	x
	r4i1p1f1	x	x	x	x	x
	r5i1p1f1		x	x		
	r6i1p1f1	x				x
KACE-1-0-G	r1i1p1f1	x	x	x		x
	r2i1p1f1	x	x	x		x
	r3i1p1f1	x	x	x		x
MIROC-ES2H	r1i1p4f2	x	x	x		x
	r2i1p4f2		x			x
	r3i1p4f2		x			x
MIROC-ES2L	r1i1p1f2	x	x	x		x
	r2i1p1f2	x	x	x		x
	r3i1p1f2	x	x	x		x
	r4i1p1f2	x	x	x		x
	r5i1p1f2	x	x	x		x
MIROC6	r1i1p1f1	x	x	x	x	x
	r2i1p1f1	x	x	x		x
	r3i1p1f1	x	x	x		x
	r4i1p1f1	x	x	x		x
	r5i1p1f1	x	x	x		x
MPI-ESM1-2-HR	r1i1p1f1	x	x	x		x
	r2i1p1f1	x	x	x		x

Supplementary Table 3: Models and their ensemble members used in this study to assess water scarcity occurrences (continued).

**Model**	**Ensemble**	**SSP1-2.6**	**SSP2-4.5**	**SSP3-7.0**	**SSP4-6.0**	**SSP5-8.5**
	r3i1p1f1			x		
	r4i1p1f1			x		
	r5i1p1f1			x		
MPI-ESM1-2-LR	r1i1p1f1	x	x	x		x
	r2i1p1f1	x	x	x		x
	r3i1p1f1	x	x	x		x
	r4i1p1f1	x	x	x		x
	r5i1p1f1	x	x	x		x
MRI-ESM2-0	r1i1p1f1	x	x	x	x	x
	r1i2p1f1					x
	r2i1p1f1	x	x	x		x
	r3i1p1f1	x	x	x		x
	r4i1p1f1	x	x	x		x
	r5i1p1f1	x	x	x		
NorESM2-LM	r1i1p1f1			x		
NorESM2-MM	r2i1p1f1		x			
TaiESM1	r1i1p1f1	x	x			
UKESM1-0-LL	r1i1p1f2	x	x	x		x
	r2i1p1f2	x	x	x		x
	r3i1p1f2	x	x	x		x
	r4i1p1f2	x	x	x		x
	r8i1p1f2		x			x
UKESM1-1-LL	r1i1p1f2	x		x		

Supplementary Table 4: Models and their ensemble members used in this study to assess water scarcity occurrences.

Model	Ensemble	SSP1-2.6	SSP2-4.5	SSP3-7.0	SSP4-6.0	SSP5-8.5
ACCESS-CM2	r1i1p1f1	x	x	x		x
	r2i1p1f1	x	x	x		x
	r3i1p1f1	x	x	x		x
	r4i1p1f1	x	x	x		x
	r5i1p1f1	x	x	x		x
ACCESS-ESM1-5	r1i1p1f1	x	x	x		x
	r2i1p1f1	x	x	x		x
	r3i1p1f1	x	x	x		x
	r4i1p1f1	x	x	x		x
	r5i1p1f1	x	x	x		x
AWI-CM-1-1-MR	r1i1p1f1	x	x	x		x
	r2i1p1f1			x		
	r3i1p1f1			x		
	r4i1p1f1			x		
	r5i1p1f1			x		
AWI-ESM-1-REcoM	r1i1p1f1	x	x			
BCC-CSM2-MR	r1i1p1f1	x	x	x		x
CAMS-CSM1-0	r1i1p1f1	x	x	x		x
	r2i1p1f1	x	x	x		x
CAS-ESM2-0	r1i1p1f1	x	x	x		x
	r3i1p1f1	x	x	x		x
CESM2-WACCM	r1i1p1f1	x	x	x		x
	r2i1p1f1		x			x
	r3i1p1f1		x			x
CESM2	r10i1p1f1	x	x	x		x
	r11i1p1f1	x				x
	r1i1p1f1	x	x	x		x
	r2i1p1f1	x	x	x		x
	r3i1p1f1		x	x		
	r4i1p1f1	x	x	x		x
CIESM	r1i1p1f1	x	x			x
CMCC-CM2-SR5	r1i1p1f1	x	x	x		x
CMCC-ESM2	r1i1p1f1	x	x	x		x
CNRM-CM6-1-HR	r1i1p1f2	x	x	x		x
CNRM-CM6-1	r1i1p1f2	x	x	x		x
	r2i1p1f2	x	x	x		x
	r3i1p1f2	x	x	x		x
	r4i1p1f2	x	x	x		x
	r5i1p1f2	x	x	x		x
CNRM-ESM2-1	r1i1p1f2	x	x	x	x	x
	r2i1p1f2	x	x	x	x	x
	r3i1p1f2	x	x	x	x	x

Supplementary Table 4: Models and their ensemble members used in this study to assess water scarcity occurrences (continued).

Model	Ensemble	SSP1-2.6	SSP2-4.5	SSP3-7.0	SSP4-6.0	SSP5-8.5
	r4i1p1f2	x	x	x	x	x
	r5i1p1f2	x	x	x	x	x
CanESM5-1	r1i1p1f1	x	x	x	x	x
	r1i1p2f1	x	x			x
	r2i1p1f1		x			x
	r2i1p2f1		x			x
	r3i1p1f1		x			x
CanESM5-CanOE	r1i1p2f1	x	x	x		x
	r2i1p2f1	x	x	x		x
	r3i1p2f1	x	x	x		x
CanESM5	r1i1p1f1	x	x	x	x	x
	r1i1p2f1	x	x	x		x
	r2i1p1f1	x	x	x	x	x
	r2i1p2f1	x	x	x		x
	r3i1p1f1	x	x	x	x	x
	r4i1p1f1				x	
	r5i1p1f1				x	
E3SM-1-1-ECA	r1i1p1f1					x
E3SM-1-1	r1i1p1f1					x
E3SM-2-0	r1i1p1f1			x		
EC-Earth3-AerChem	r1i1p1f1			x		
	r3i1p1f1			x		
EC-Earth3-CC	r1i1p1f1		x			x
	r4i1p1f1		x			
	r6i1p1f1		x			
	r7i1p1f1		x			
	r8i1p1f1		x			
EC-Earth3-Veg-LR	r1i1p1f1	x	x	x		x
	r2i1p1f1	x	x	x		x
	r3i1p1f1	x	x	x		x
EC-Earth3-Veg	r12i1p1f1			x		
	r1i1p1f1	x	x	x	x	x
	r2i1p1f1	x	x	x		x
	r3i1p1f1	x	x	x		x
	r4i1p1f1	x	x	x		x
	r6i1p1f1	x	x			x
EC-Earth3	r1i1p1f1	x	x	x		x
	r2i1p1f1		x			
	r3i1p1f1					x
	r4i1p1f1	x	x	x		x
	r5i1p1f1	x	x	x		
	r6i1p1f1	x	x	x		x

Supplementary Table 4: Models and their ensemble members used in this study to assess water scarcity occurrences (continued).

Model	Ensemble	SSP1-2.6	SSP2-4.5	SSP3-7.0	SSP4-6.0	SSP5-8.5
	r9i1p1f1	x		x		x
FGOALS-f3-L	r1i1p1f1	x	x	x		x
FGOALS-g3	r1i1p1f1	x	x	x	x	x
	r2i1p1f1	x	x	x		x
	r3i1p1f1	x	x	x		x
	r4i1p1f1	x	x	x		x
	r5i1p1f1			x		
FIO-ESM-2-0	r1i1p1f1	x	x			x
	r2i1p1f1	x	x			x
	r3i1p1f1	x	x			x
GFDL-CM4	r1i1p1f1		x			x
GFDL-ESM4	r1i1p1f1	x	x	x		x
GISS-E2-1-G	r1i1p3f1	x	x	x	x	x
	r1i1p5f1	x	x	x	x	x
	r2i1p3f1	x	x	x		x
	r2i1p5f1	x	x	x		x
	r3i1p3f1	x	x	x		x
GISS-E2-1-H	r1i1p3f1	x		x	x	x
	r2i1p3f1	x	x			x
	r3i1p3f1	x	x			x
	r4i1p3f1	x	x			x
	r5i1p3f1	x	x			x
GISS-E2-2-G	r1i1p3f1	x	x	x		x
	r2i1p3f1	x	x	x		x
	r3i1p3f1	x	x	x		x
	r4i1p3f1	x	x	x		x
	r5i1p3f1	x	x	x		x
HadGEM3-GC31-LL	r1i1p1f3	x	x			x
	r2i1p1f3		x			x
	r3i1p1f3		x			x
	r4i1p1f3		x			x
	r5i1p1f3		x			
HadGEM3-GC31-MM	r1i1p1f3	x				x
	r2i1p1f3					x
	r3i1p1f3					x
	r4i1p1f3					x
IITM-ESM	r1i1p1f1	x	x			x
INM-CM4-8	r1i1p1f1	x	x	x		x
INM-CM5-0	r1i1p1f1	x	x	x		x
	r2i1p1f1			x		
	r3i1p1f1			x		
	r4i1p1f1			x		

Supplementary Table 4: Models and their ensemble members used in this study to assess water scarcity occurrences (continued).

**Model**	**Ensemble**	**SSP1-2.6**	**SSP2-4.5**	**SSP3-7.0**	**SSP4-6.0**	**SSP5-8.5**
	r5i1p1f1			x		
IPSL-CM6A-LR	r1i1p1f1	x	x	x	x	x
	r2i1p1f1	x	x	x	x	x
	r3i1p1f1	x	x	x	x	x
	r4i1p1f1	x	x	x	x	x
	r5i1p1f1		x	x		
	r6i1p1f1	x			x	x
KACE-1-0-G	r1i1p1f1	x	x	x		x
	r2i1p1f1	x	x	x		x
	r3i1p1f1	x	x	x		x
MIROC-ES2H	r1i1p4f2	x	x			
	r2i1p4f2		x			
	r3i1p4f2		x			
MIROC-ES2L	r1i1p1f2	x	x	x		x
	r2i1p1f2	x	x	x		x
	r3i1p1f2	x	x	x		x
	r4i1p1f2	x	x	x		x
	r5i1p1f2	x	x	x		x
MIROC6	r1i1p1f1	x	x	x	x	x
	r2i1p1f1	x	x	x		x
	r3i1p1f1	x	x	x		x
	r4i1p1f1	x	x	x		x
	r5i1p1f1	x	x	x		x
MPI-ESM1-2-HR	r1i1p1f1	x	x	x		x
	r2i1p1f1	x	x	x		x
	r3i1p1f1			x		
	r4i1p1f1			x		
	r5i1p1f1			x		
MPI-ESM1-2-LR	r1i1p1f1	x	x	x		x
	r2i1p1f1	x	x	x		x
	r3i1p1f1	x	x	x		x
	r4i1p1f1	x	x	x		x
	r5i1p1f1	x	x	x		x
MRI-ESM2-0	r1i1p1f1	x	x	x	x	x
	r1i2p1f1					x
	r2i1p1f1	x	x	x		x
	r3i1p1f1	x	x	x		x
	r4i1p1f1	x	x	x		x
	r5i1p1f1	x	x	x		
NESM3	r1i1p1f1	x	x			x
	r2i1p1f1	x	x			x
NorESM2-LM	r1i1p1f1	x	x	x		x

Supplementary Table 4: Models and their ensemble members used in this study to assess water scarcity occurrences (continued).

Model	Ensemble	SSP1-2.6	SSP2-4.5	SSP3-7.0	SSP4-6.0	SSP5-8.5
	r1i1p4f1		x			
	r2i1p1f1		x			
	r3i1p1f1		x			
NorESM2-MM	r1i1p1f1	x	x	x		x
	r2i1p1f1		x			
TaiESM1	r1i1p1f1	x	x	x		x
UKESM1-0-LL	r1i1p1f2	x	x	x		x
	r2i1p1f2	x	x	x		x
	r3i1p1f2	x	x	x		x
	r4i1p1f2	x	x	x		x
	r8i1p1f2	x	x	x		x
UKESM1-1-LL	r1i1p1f2	x		x		

Supplementary References

[1] Iturbide, M., Gutiérrez, J.M., Alves, L.M., Bedia, J., Cerezo-Mota, R., Cimadevilla, E., : An update of IPCC climate reference regions for subcontinental analysis of climate model data: definition and aggregated datasets. Earth System Science Data 12(4), 2959–2970 (2020) 10.5194/essd-12-2959-2020

[2] Wang, T., Sun, F.: Global gridded gdp data set consistent with the shared socioeconomic pathways. Scientific Data **9**(1) (2022) 10.1038/s41597-022-01300-x
